# Supplementary material for: MAPK inhibitor sensitivity scores predict sensitivity driven by the immune infiltration in pediatric low-grade gliomas
Source: Nat Commun. 2023 Jul 27;14:4533. doi: 10.1038/s41467-023-40235-8 (PMC10374577; doi:10.1038/s41467-023-40235-8)
Supplement: Supplementary file 1 — Supplementary Information [file 41467_2023_40235_MOESM1_ESM.pdf]

# MAPK inhibitor sensitivity scores predict sensitivity driven by the immune infiltration in pediatric low-grade gliomas

Romain Sigaud [1,2,3*], Thomas K. Albert [4], Caroline Hess [1,2,3,5], Thomas Hielscher [6], Nadine Winkler [1,2,3,7], Daniela Kocher [1,2,3,7], Carolin Walter [8], Daniel Münter [4], Florian Selt [1,2,3,9], Diren Usta [1,2,3,9], Jonas Ecker [1,2,3,9], Angela Brentrup [10], Martin Hasselblatt [11], Christian Thomas [11], Julian Varghese [8], David Capper [12,13], Ulrich W. Thomale [14], Pablo Hernáiz Driever [15], Michèle Simon [15], Svea Horn [15], Nina Annika Herz [15], Arend Koch [13], Felix Sahm [16,17], Stefan Hamelmann [16,17], Augusto Faria-Andrade [18], Nada Jabado [18,19,20], Martin U Schuhmann [21], Antoinette YN Schouten-van Meeteren [22], Eelco Hoving [22], Tilman Brummer [23], Cornelis M. van Tilburg [1,2,3,9], Stefan M. Pfister [1,3,9,24], Olaf Witt [1,2,3,9], David T.W. Jones [1,25], Kornelius Kerl [4], Till Milde [1,2,3,9*]

**SUPPLEMENTARY INFORMATION**

## SUPPLEMENTARY ANALYSIS

**Related to *MSSs are not confounded and are elevated in tumor-associated microglia***

**PDX-derived MSS**

Since our MSSs were derived from cell lines, i.e. without microenvironmental cells, we verified whether key genes related to sensitivity in the microenvironment cells might be missing and could be included in order to improve the MSSs prediction power. To alleviate the lack of data from PDX model recapitulating the true pLGG biology,72 and lack of large dataset derived from patient data with known response to MAPKi therapy, we generated PDX-derived MSS for the BRAFi Type I½ encorafenib, and the MEKi trametinib and binimetinib using the XevaDB PDX-dataset. We first show that the PDX samples used in the MEKi cohorts (binimetinib, trametininb) retains a significant proportion of immune cells (probably from the primary material the PDX is derived from), while the PDX samples used in the BRAFi Type I½ cohort (encorafenib) retained a significantly smaller proportion of immune cells (Supplementary Fig. S24A). We could generate PDX-derived sensitivity gene signatures for encorafenib (Enco-PDX MSS), binimetinib (Bini-PDX MSS) and trametinib (Tram-PDX MSS) by selecting the genes contributing to the enrichment edge of the “Hallmark_KRAS_SIGNALING_UP” gene signature in the responsive samples (tumor volume reduction upon treatment) compared to the non-responsive samples (tumor volume increase upon treatment). There was only a small overlap between the genes comprised in the PDX-derived MSS and those comprised in our original MSS (Supplementary Fig. S24B, Supplementary Data S4). We then applied these new signatures in our pLGG patient datasets. In the pLGG samples treated with the MEKi trametinib, the Tram-PDX MSS correlated with treatment response in a similar than our MEK1/2i MSS (Supplementary Fig. S24C and Fig. 4D, respectively). The Bini-PDX MSS poorly predicted treatment response (Supplementary Fig. S19D), and had a similar pattern than the predicted immune infiltration (Supplementary Fig S22). In the cohort of melanoma samples with mutually exclusive BRAF fusion alterations and treated with the BRAFi Type I½ vemurafenib, the Enco-PDX MSS did not outperform our cell line-derived MSS (Supplementary Fig. S24E). Taken together, the data suggest that our MSS signatures contains enough genes to be able to predict MAPKi sensitivity with high enough efficacy in patient samples, and that adding genes specific to a certain cell population has the risk of potentially introducing a confounding effect.

# SUPPLEMENTARY FIGURES

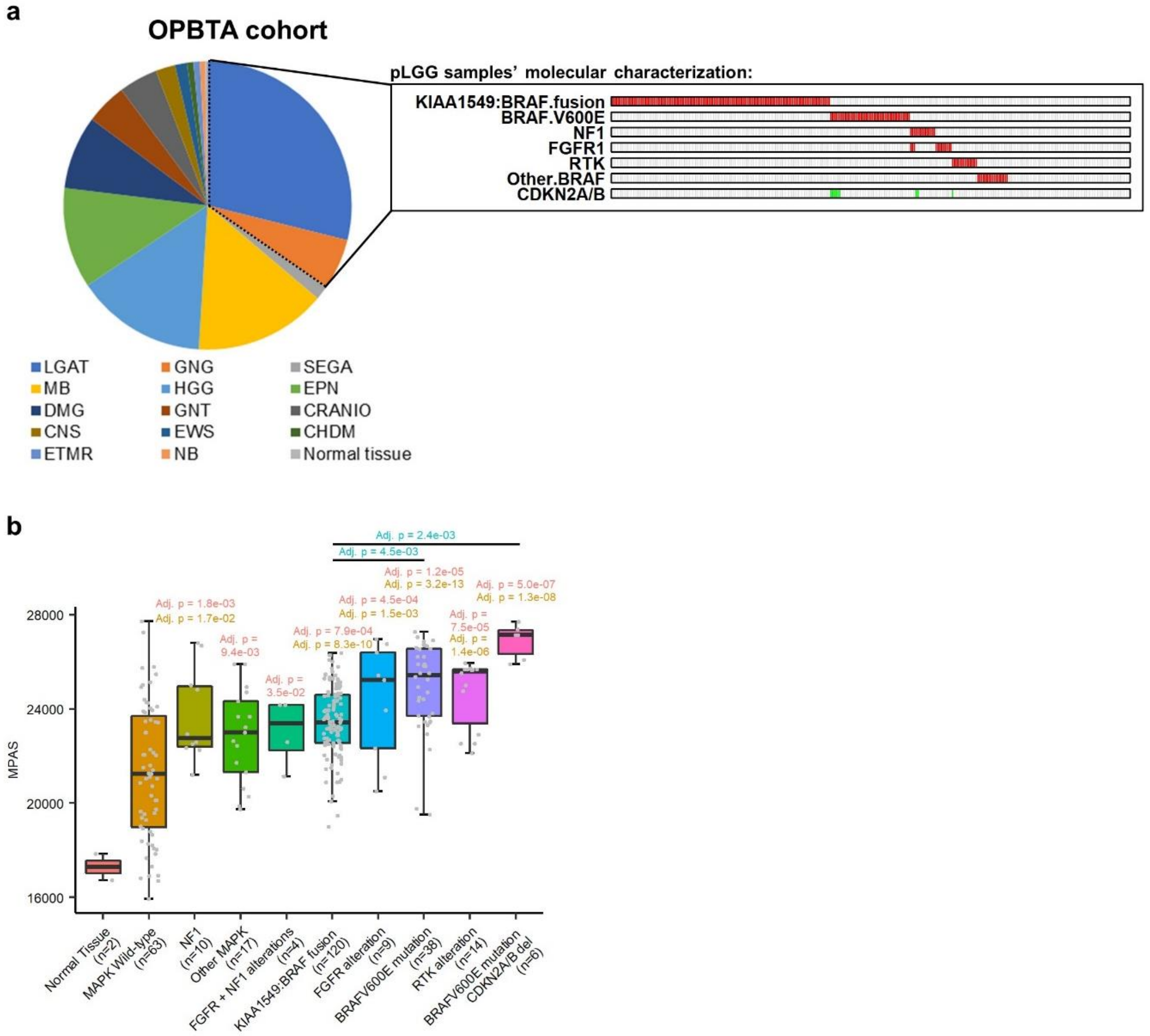


**Supplementary Fig. S1: Cohort description and MPAS in the OPBTA dataset**

**a**, Description of the Open Pediatric Brain Tumor Atlas (OPBTA) cohort, regrouping several primary pediatric brain tumor samples from different entities and MAPK alteration backgrounds. LGAT: low-grade glioma/astrocytoma; GNG: ganglioglioma; SEGA: Subependymal Giant Cell Astrocytoma; MB: medulloblastoma; HGG: high-grade glioma; EPN: ependymoma; DMG: diffuse midline glioma; GNT: glial neuronal tumor; CRANIO: craniopharyngioma; CNS: other CNS embryonal tumor; EWS: Ewin Sarcoma; CHDM: chordoma; ETMR: embryonal tumor with multilayer rosettes; NB = neuroblastoma; normal tissue = epilepsy and connective tissue. **b**, boxplots depicting the MPAS in all LGG molecular subtypes from the OPBTA cohort. Data from n = 283 biologically independent samples were used. Boxplots depict the median, first and third quartiles. Whiskers extend from the hinge to the largest/smallest value no further than 1.5 * IQR from the hinge (where IQR is the inter-quartile range).

One-way ANOVA followed by the Tukey's ‘Honest Significant Difference’ was used to measure significance. Significance is reported color-coded based on the group of reference (normal tissue = “rose”, MAPK wild-type = “gold”, KIAA1549:BRAFfusion = “dark teal”). Source data are provided as a Source Data file.

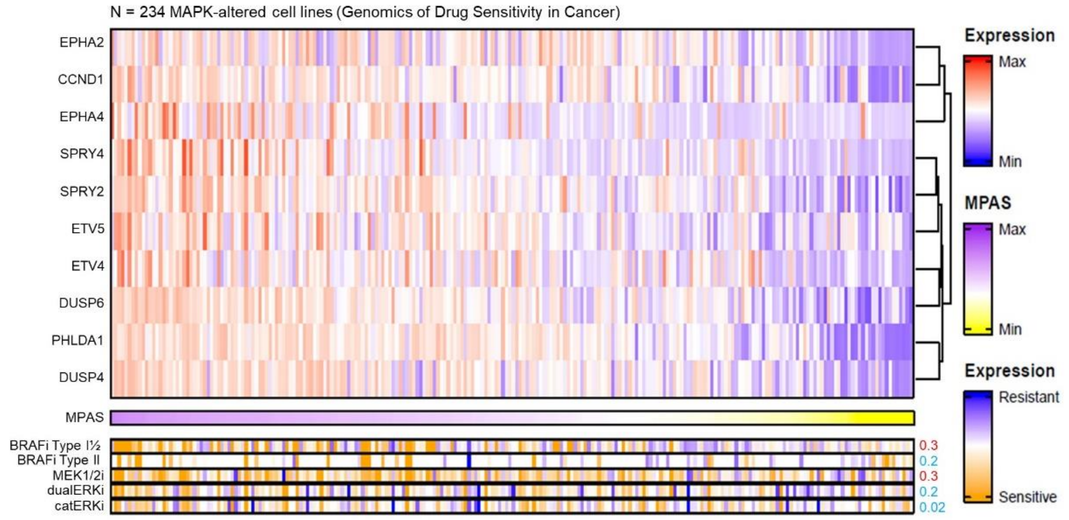


**Supplementary Fig. S2: MPAS correlation with MAPKi measured sensitivity in the GDSC dataset**

The top heatmap depicts the gene expression level of the MPAS genes in the 234 MAPK-altered cell lines from the GDSC dataset. The middle heatmap depicts the MPAS as measured by ssGSEA (samples are ordered by decreasing MPAS). Bottom heatmap depicts MAPKi sensitivity for each class. The median IC50 z-score across all drugs belonging to the same class was used a MAPKi sensitivity score for a given class. On the right-side is indicated the coefficient of correlation between MPAS and MAPKi sensitivity. Source data are provided as a Source Data file.

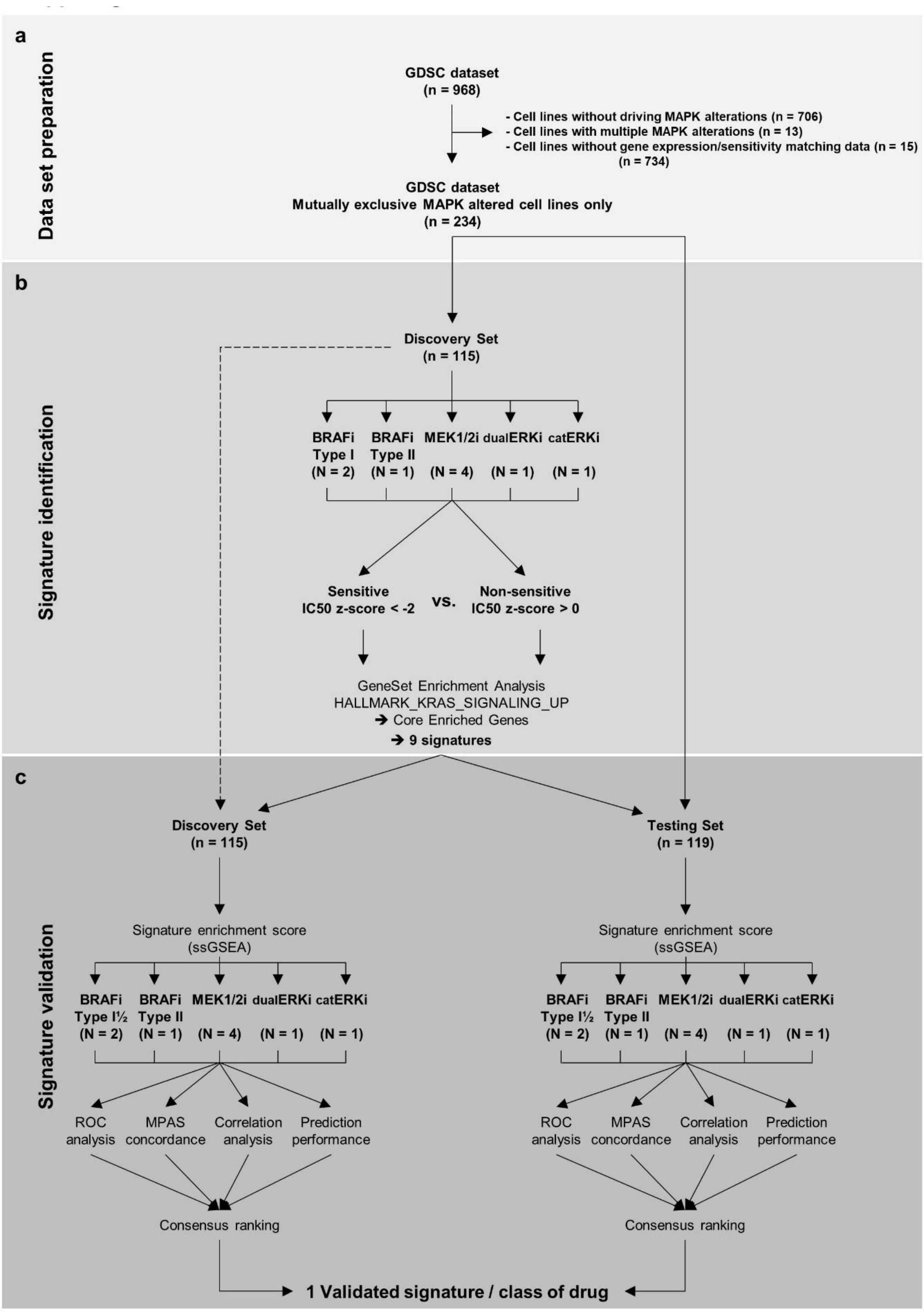
a
Data set preparation
GDSC dataset
(n = 968)
- Cell lines without driving MAPK alterations (n = 706)
- Cell lines with multiple MAPK alterations (n = 13)
- Cell lines without gene expression/sensitivity matching data (n = 15)
(n = 734)
GDSC dataset
Mutually exclusive MAPK altered cell lines only
(n = 234)
b
Signature identification
Discovery Set
(n = 115)
BRAFi Type I (N = 2)
BRAFi Type II (N = 1)
MEK1/2i (N = 4)
dualERKi (N = 1)
catERKi (N = 1)
Sensitive
IC50 z-score < -2
vs.
Non-sensitive
IC50 z-score > 0
GeneSet Enrichment Analysis
HALLMARK_KRAS_SIGNALING_UP
➔ Core Enriched Genes
➔ 9 signatures
c
Signature validation
Discovery Set
(n = 115)
Testing Set
(n = 119)
Signature enrichment score
(ssGSEA)
BRAFi Type I½ (N = 2)
BRAFi Type II (N = 1)
MEK1/2i (N = 4)
dualERKi (N = 1)
catERKi (N = 1)
ROC analysis
MPAS concordance
Correlation analysis
Prediction performance
Consensus ranking
Signature enrichment score
(ssGSEA)
BRAFi Type I½ (N = 2)
BRAFi Type II (N = 1)
MEK1/2i (N = 4)
dualERKi (N = 1)
catERKi (N = 1)
ROC analysis
MPAS concordance
Correlation analysis
Prediction performance
Consensus ranking
1 Validated signature / class of drug

**Supplementary Fig. S3: Gene signatures identification pipeline.**

**a**, cell lines from the GDSC dataset with paired gene expression and MAPKi sensitivity was sorted based on MAPK alteration status. **b**, the dataset was then split into Discovery and Testing sets. The Discovery set was used to generate sensitivity signatures (n = 9). **c**, the signatures were then validated using the Testing set. ssGSEA scores were used to measure signature scores, and metrics derived from ROC analysis, correlation with MAPKi sensitivity, concordance with MAPK pathway activity and prediction efficiency were ranked in both Discovery and Testing sets in order to identify the best performing signatures for a given class of MAPKi. “n” indicates number of cell lines, while “N” indicates number of drugs comprised in each MAPKi class.

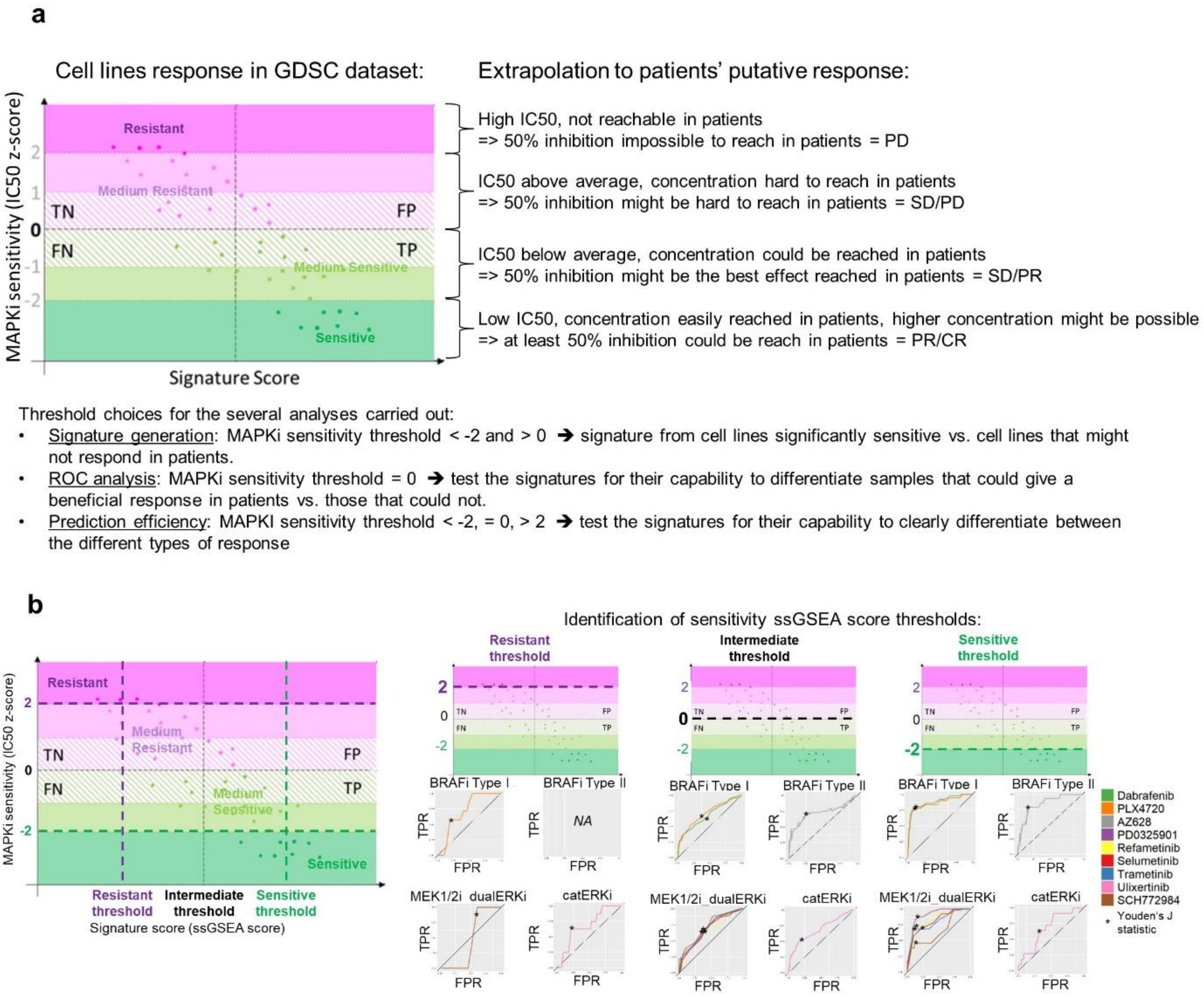


**Supplementary Fig. S4: Supplementary information on sensitivity threshold determination**

**a**, a comprehensive illustration of the rational used to select thresholds throughout the analysis is also depicted. **b**, a comprehensive illustration of the rational used to select ssGSEA thresholds for each sensitivity levels (sensitive, intermediate, resistant). A ROC analysis was performed on the GDSC dataset, fixing the IC50 z-score threshold at 2, 0 and -2, respectively, and selection of the best ssGSEA threshold was done based on the Youden's J statistic. PD: progressive disease, SD: stable disease, PR: partial response, CR: complete remission, TN: true negative, TP: true positive, FP: false positive, FN: false negative, TPF: true positive rate, FPR: false positive rate, NA: not applicable.

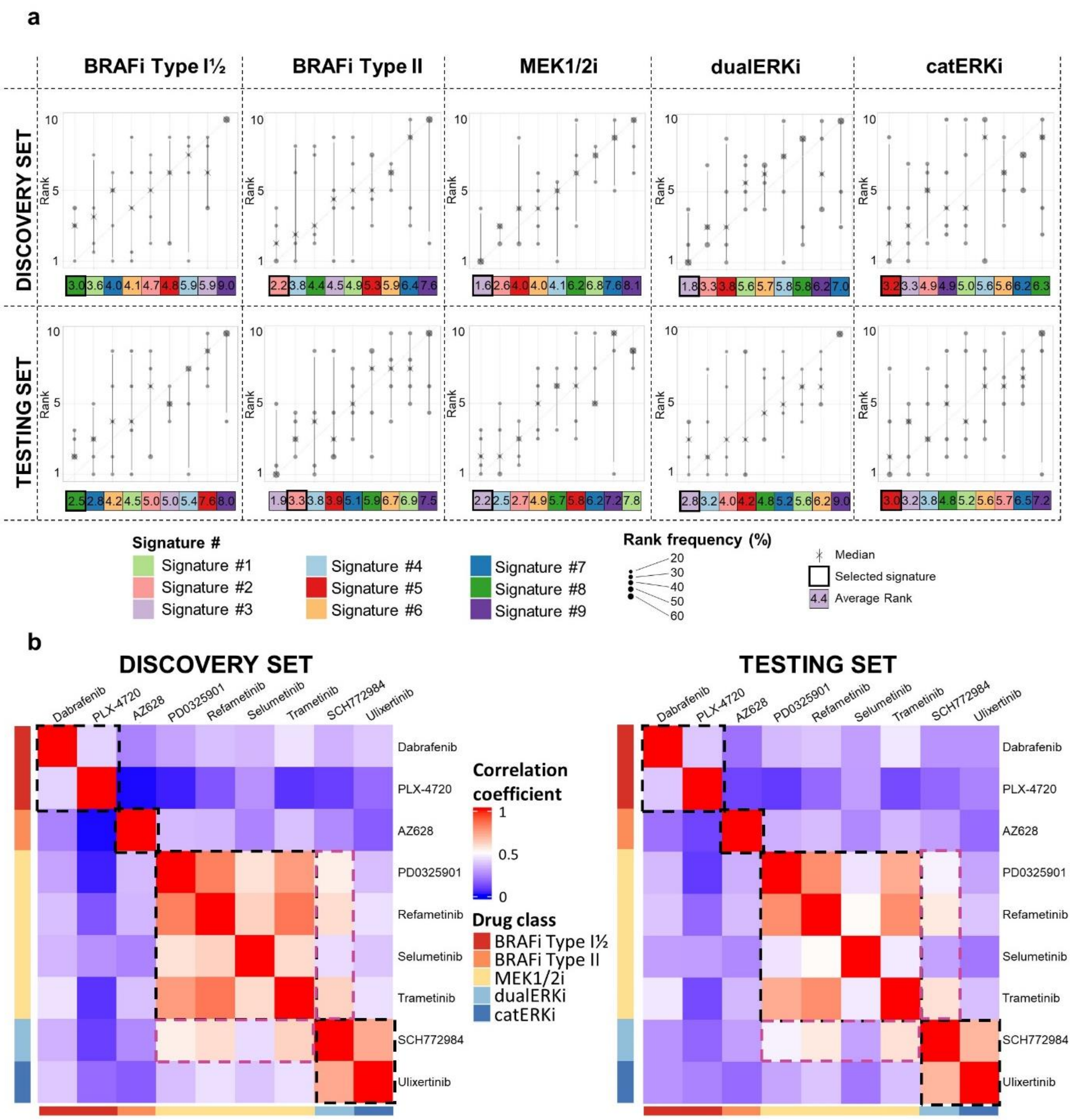


**Supplementary Fig. S5: Consensus ranking details and MAPKi sensitivity overlap**

**a**, a detailed summary of each signatures' performance following consensus ranking across metrics and MAPKi type is shown. Signatures are color-coded (squares), and the area of each blob at position (Ai, rank j) is proportional to the relative frequency Ai achieved rank j across multiple metrics (e.g. F1-score, Youden's J stat etc.). The median rank for each algorithm is indicated by a black cross. The average rank across all metrics is reported in the squares below the graph. Data from n = 115 (discovery) and n = 119 (testing) independent cells were used. **b**, the coefficient of correlation estimating the correlation between MAPKi sensitivity to a given MAPKi and sensitivity to another MAPKi is shown. Black dashed rectangles groups MAPKi belonging to the same class. Purple dashed

rectangles highlight the overlap of sensitivity to MEK1/2i and dualERKi. Source data are provided as a Source Data file.

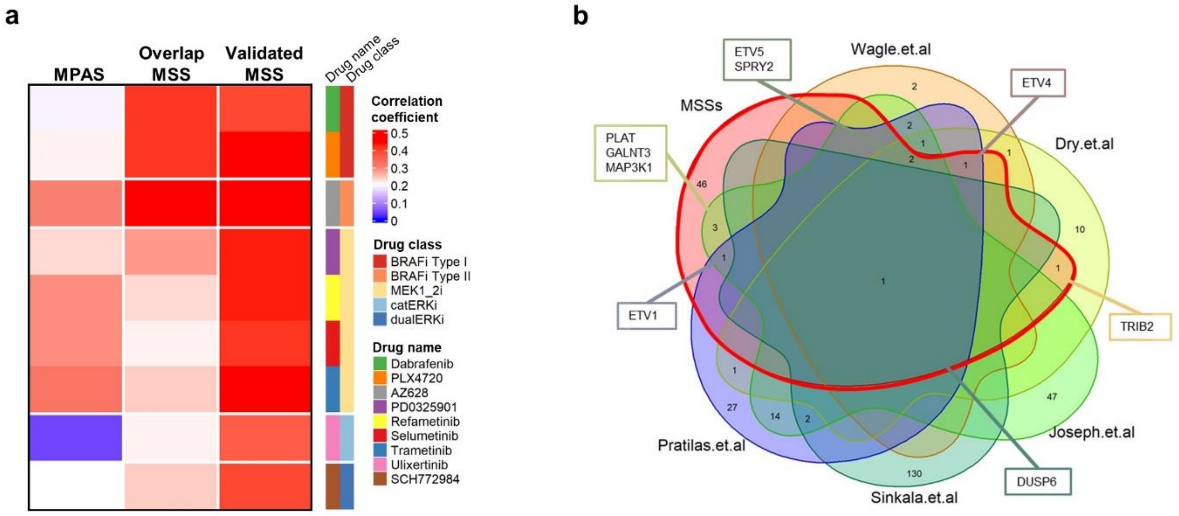


**Supplementary Fig. S6: MPAS/MSS/overlap-MSS correlation with MAPKi measured sensitivity in the GDSC dataset and MSS signatures overlap with existing MAPKi signatures**

**a**, heatmap depicting Pearson's coefficient of correlation between MPAS/Overlap MSS/Validated MSS ssGSEA scores and MAPKi sensitivity (IC50 z-score) in the cell lines from the GDSC dataset. **b**, Venn diagram depicting genes overlap between MSS signatures and signatures already described as being involved in MAPK pathway activity and/or MAPKi sensitivity. Source data are provided as a Source Data file.

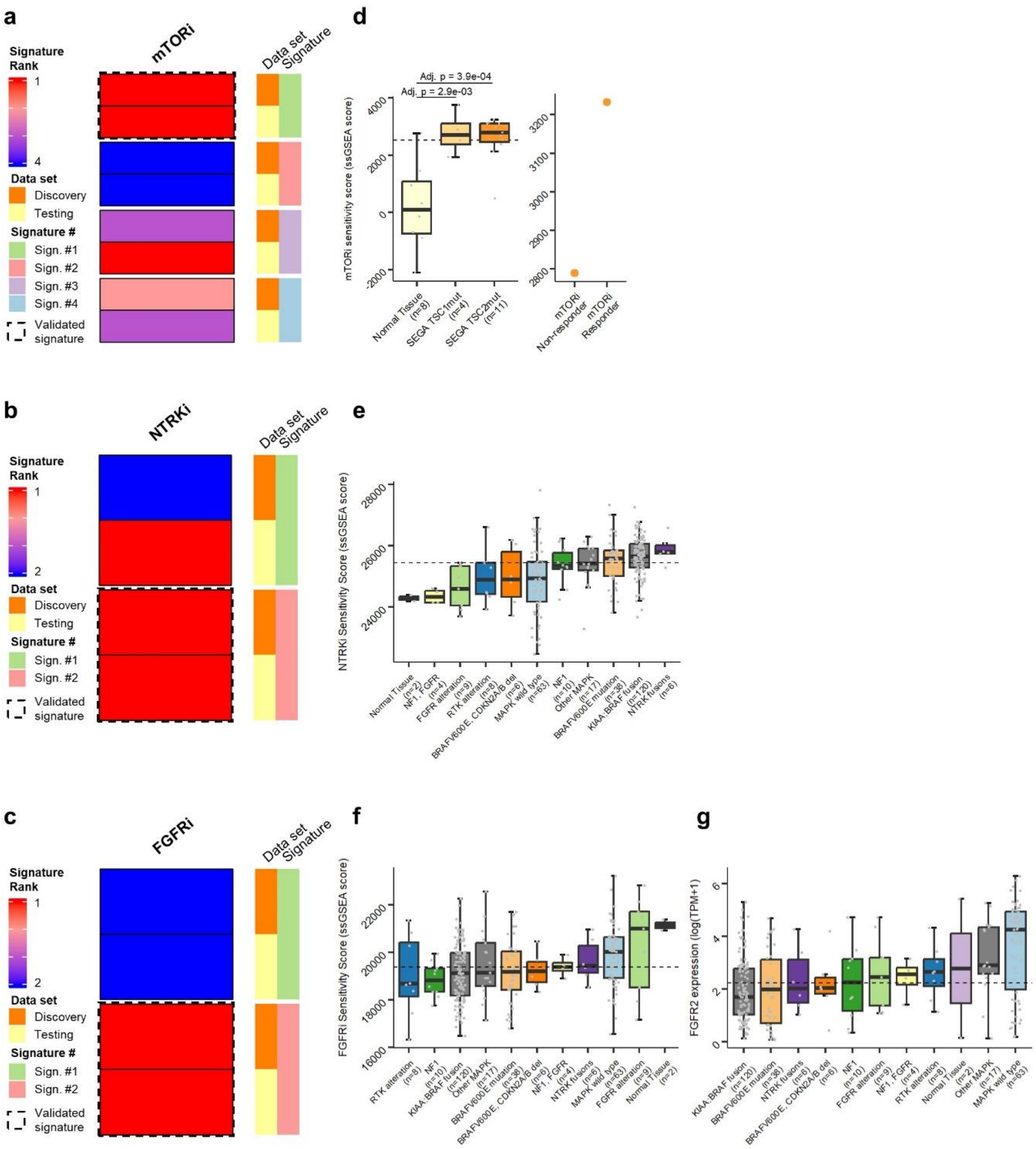


**Supplementary Fig. S7: Validation of the gene signature pipeline by generating gene expression-derived signature for other pLGG relevant drug classes: NTRKi, FGFRi, mTORi.**

**a-c**, heatmap depicting the final consensus ranking for the signatures based on their ability to best predict sensitivity to mTOR inhibitors (**a**), NTRK inhibitors (**b**), and FGFR inhibitor (**c**). **d**, boxplots depicting the ssGSEA predictive sensitivity score to mTORi in TSC1/2 mutated primary samples and normal samples from Bongaarts et al.. Data from n = 23 biologically independent samples were used. Is also shown the ssGSEA predictive sensitivity score to mTORi in TSC1/2 mutated primary samples with known response to mTOR inhibitor therapy (n = 2 biologically independent samples). **e-f**, boxplots

depicting the ssGSEA predictive sensitivity score to NTRKi (**e**), FGFRI (**f**) for each pLGG molecular subtype in the OPBTA. Data from n = 283 biologically independent samples were used. **g**, boxplots depicting the gene expression level (log2(TPM+1) of FGFR2 in each pLGG molecular subtype. Data from n = 283 biologically independent samples were used. Boxplots depict the median, first and third quartiles. Whiskers extend from the hinge to the largest/smallest value no further than 1.5 * IQR from the hinge (where IQR is the inter-quartile range). One-way ANOVA followed by the Tukey's ‘Honest Significant Difference’ was used to measure significance. Source data are provided as a Source Data file.

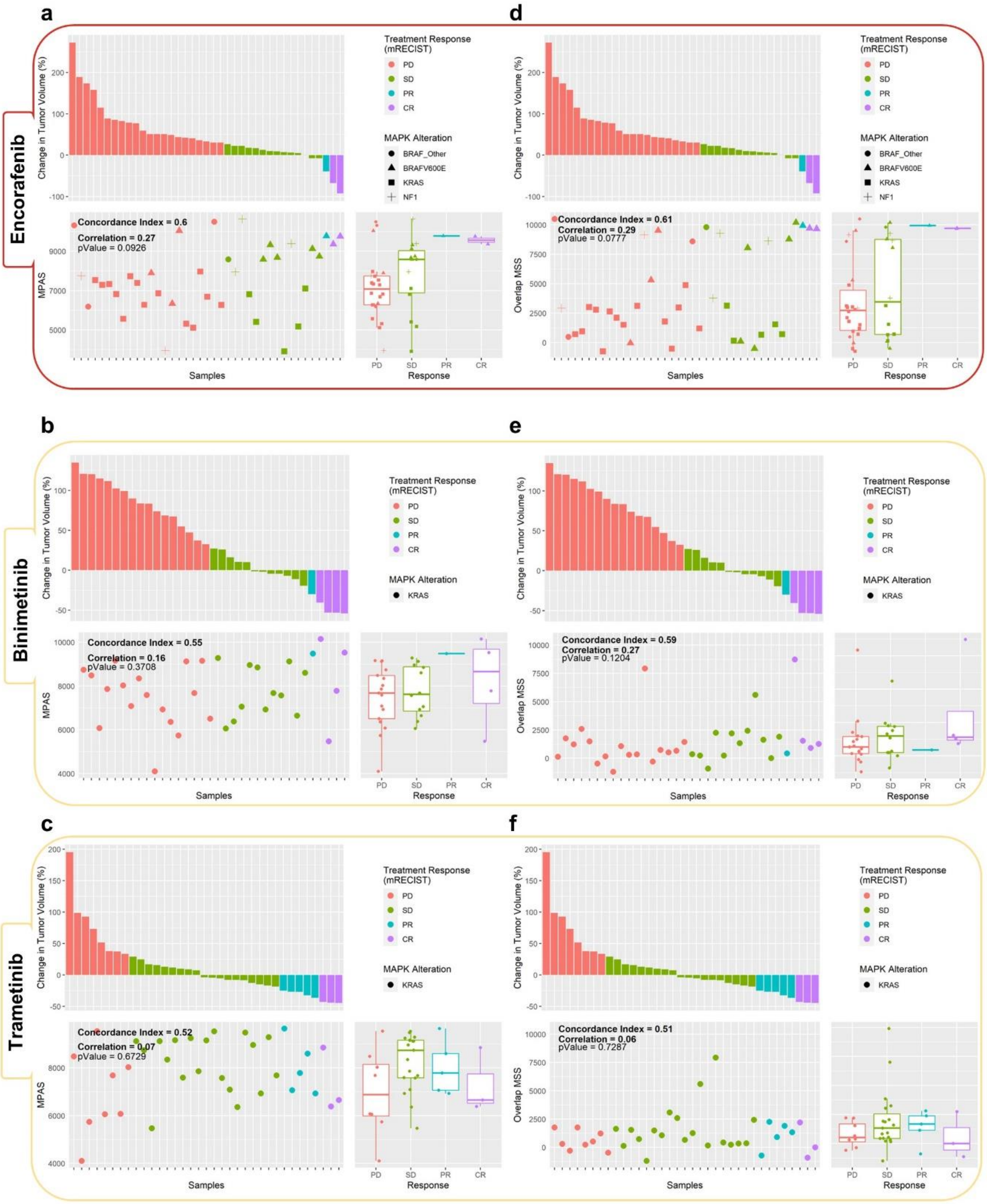


**Supplementary Fig. S8: Performance of the MPAS and the Overlap MSS in the PDX trial dataset**

The MPAS and the Overlap MSS derived from the common genes across our validated MSS signatures were tested and challenged. **a-c**, the performance of the Overlap MSS to predict MAPKi sensitivity was tested in the PDX cohort. **d-f**, MPAS performance to predict MAPKi sensitivity in the PDX dataset was also estimated. Waterfall plots were used to depict MAPKi treatment response as measured in the original publication (i.e. primary response as described in the original publication;

mRECIST criteria) for each samples, and dotplots were used to depict corresponding MAPKi sensitivity scores. Samples were grouped based on treatment response in boxplots. This tryptic analysis was done for PDX treated with the BRAFi type I ½ encorafenib, MEK1/2i trametinib and binimetinib. Significance was calculated using a two-tailed t-test for the correlation analysis, and one-way ANOVA followed by the Tukey's 'Honest Significant Difference' in the boxplots. Boxplots depict the median, first and third quartiles. Whiskers extend from the hinge to the largest/smallest value no further than 1.5 * IQR from the hinge (where IQR is the inter-quartile range). Difference was not significant if not specified. Data from n = 39, 34 and 35 biologically independent animals were used in the encorafenib, binimetinib and trametinib cohorts, respectively. Source data are provided as a Source Data file.

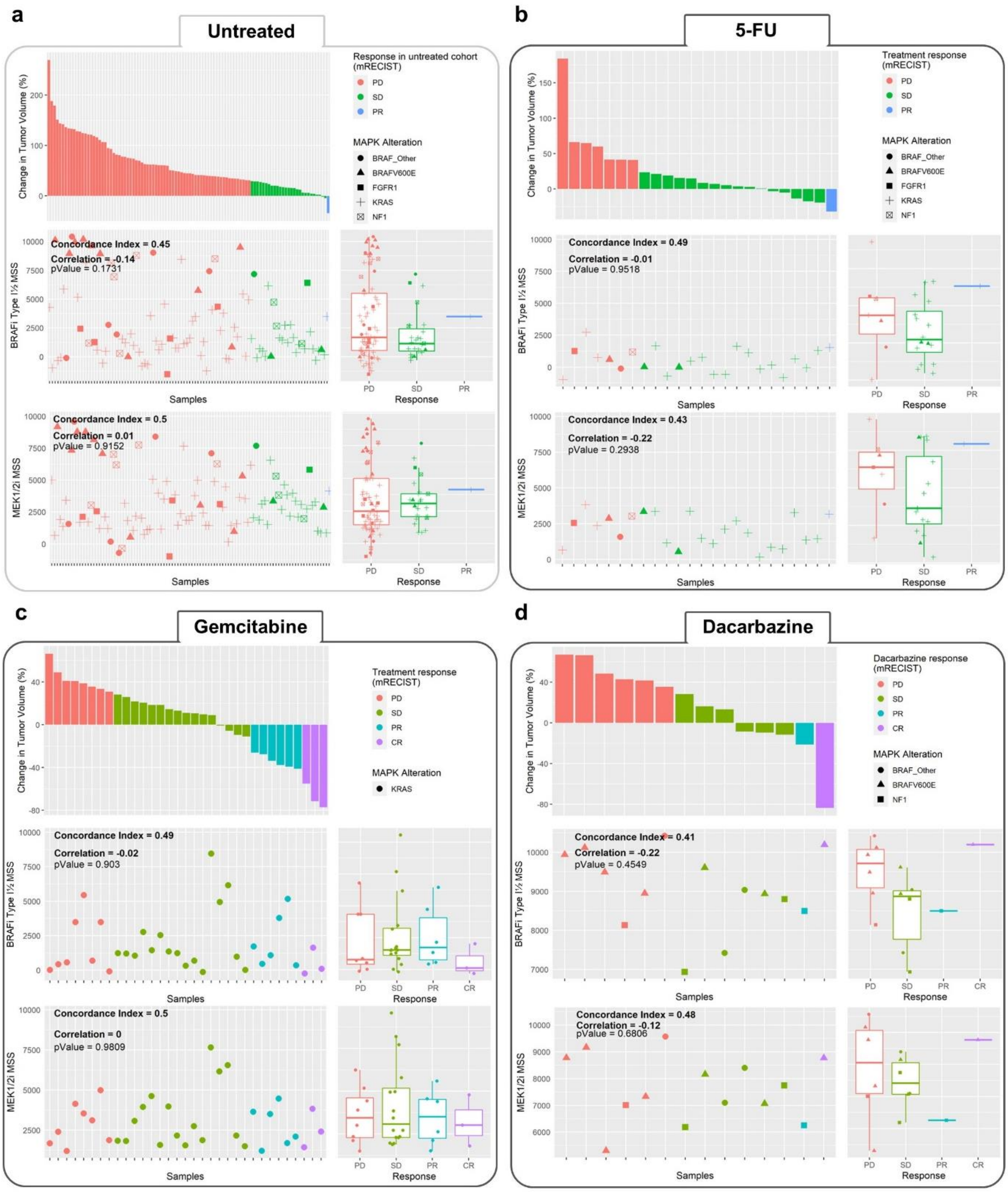


**Supplementary Fig. S9: Performance of the BRAFi Type I ½ and MEK1/2i signatures in the PDX samples untreated, or treated with chemotherapy**

The BRAFi type I ½ and MEK1/2i sensitivity signatures were tested in cohorts untreated or treated with chemotherapeutic agents. **a**, signatures' performance to predict response in the untreated mice from the PDX dataset. **b-d**, signatures' performance to predict response in mice treated with chemotherapeutic agents. Waterfall plots were used to depict MAPKi treatment response as measured

in the original publication (i.e. primary response as described in the original publication; mRECIST criteria) for each samples, and dotplots were used to depict corresponding MAPKi sensitivity scores. Samples were grouped based on treatment response in boxplots. This tryptic analysis was done for PDX treated with the BRAFi type I ½  encorafenib, MEK1/2i trametinib and binimetinib. Significance was calculated using a two-tailed t-test for the correlation analysis, and one-way ANOVA followed by the Tukey's ‘Honest Significant Difference’ in the boxplots. Boxplots depict the median, first and third quartiles. Whiskers extend from the hinge to the largest/smallest value no further than 1.5 * IQR from the hinge (where IQR is the inter-quartile range). Difference was not significant if not specified. Data from n = 100, 24, 33 and 14 biologically independent animals were used in the untreated, 5-FU, gemcitabine and dacarbazine cohorts, respectively. Source data are provided as a Source Data file.

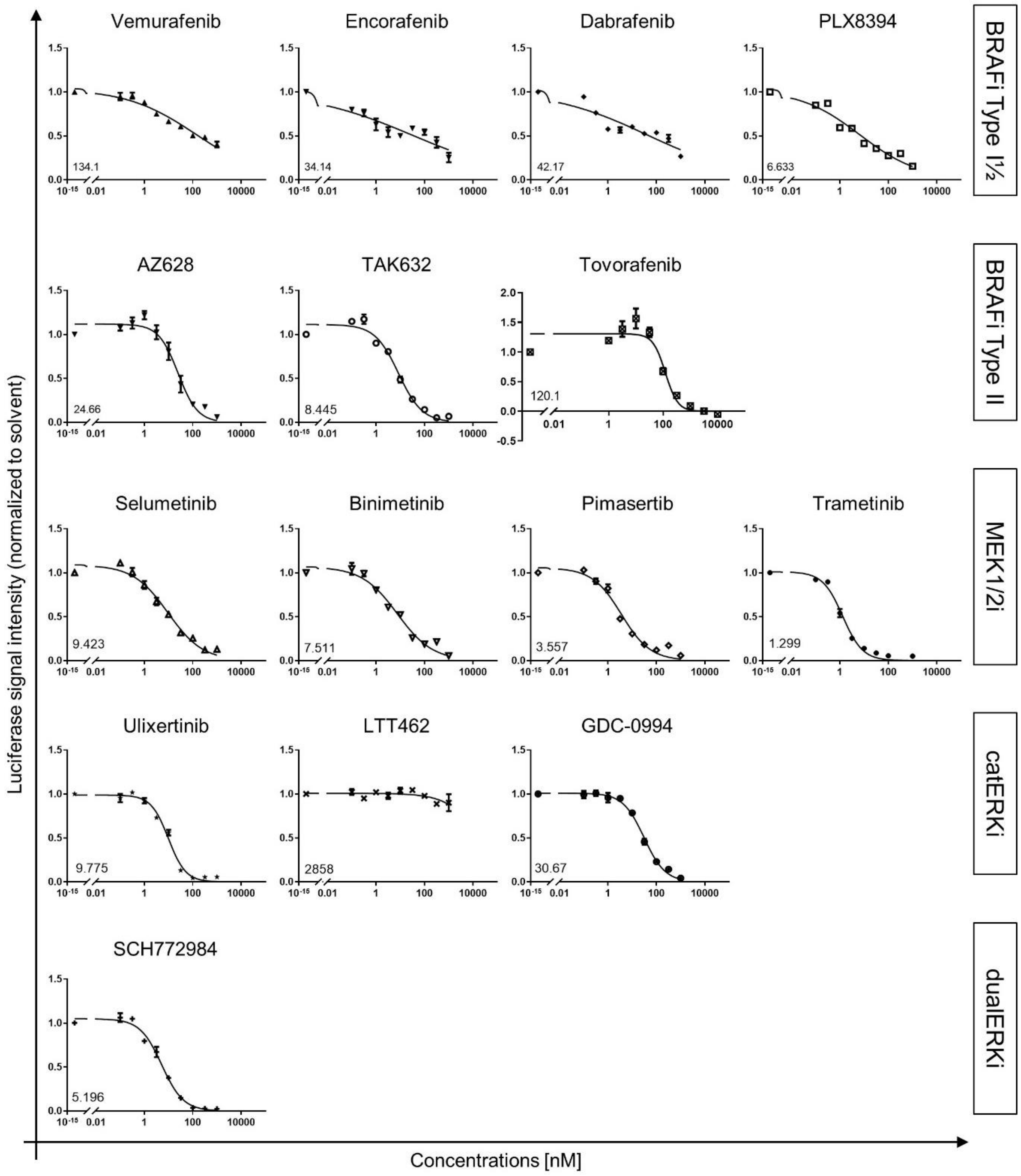


**Supplementary Fig. S10: MAPKi mini-screen for IC50 calculation in DKFZ-BT314 transduced with the pDIPZ reporter**

Dose-response curves of MAPKi belong to the BRAFi Type I ½, BRAFi Type II, MEK1/2i, catERKi and dualERKi classes. $IC_{50}$ were measured in technical duplicates for three independent biological replicates. Data are presented as mean values +/- SD. Source data are provided as a Source Data file.

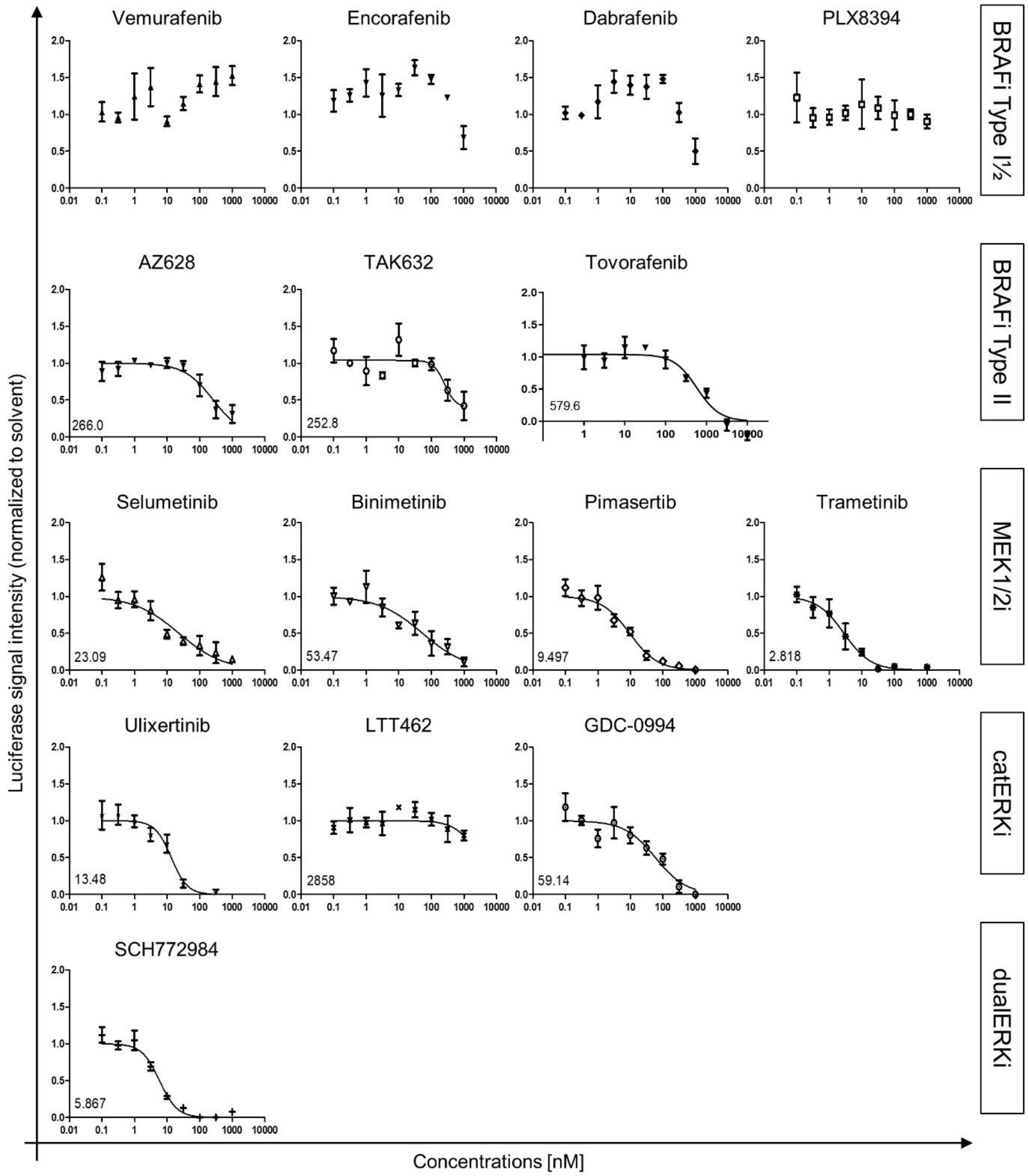


**Supplementary Fig. S11: MAPKi mini-screen for IC50 calculation in DKFZ-BT317 transduced with the pDIPZ reporter**

Dose-response curves of MAPKi belong to the BRAFi Type I ½, BRAFi Type II, MEK1/2i, catERKi and dualERKi classes. $IC_{50}$ were measured in technical duplicates for three independent biological replicates. Data are presented as mean values +/- SD. Source data are provided as a Source Data file.

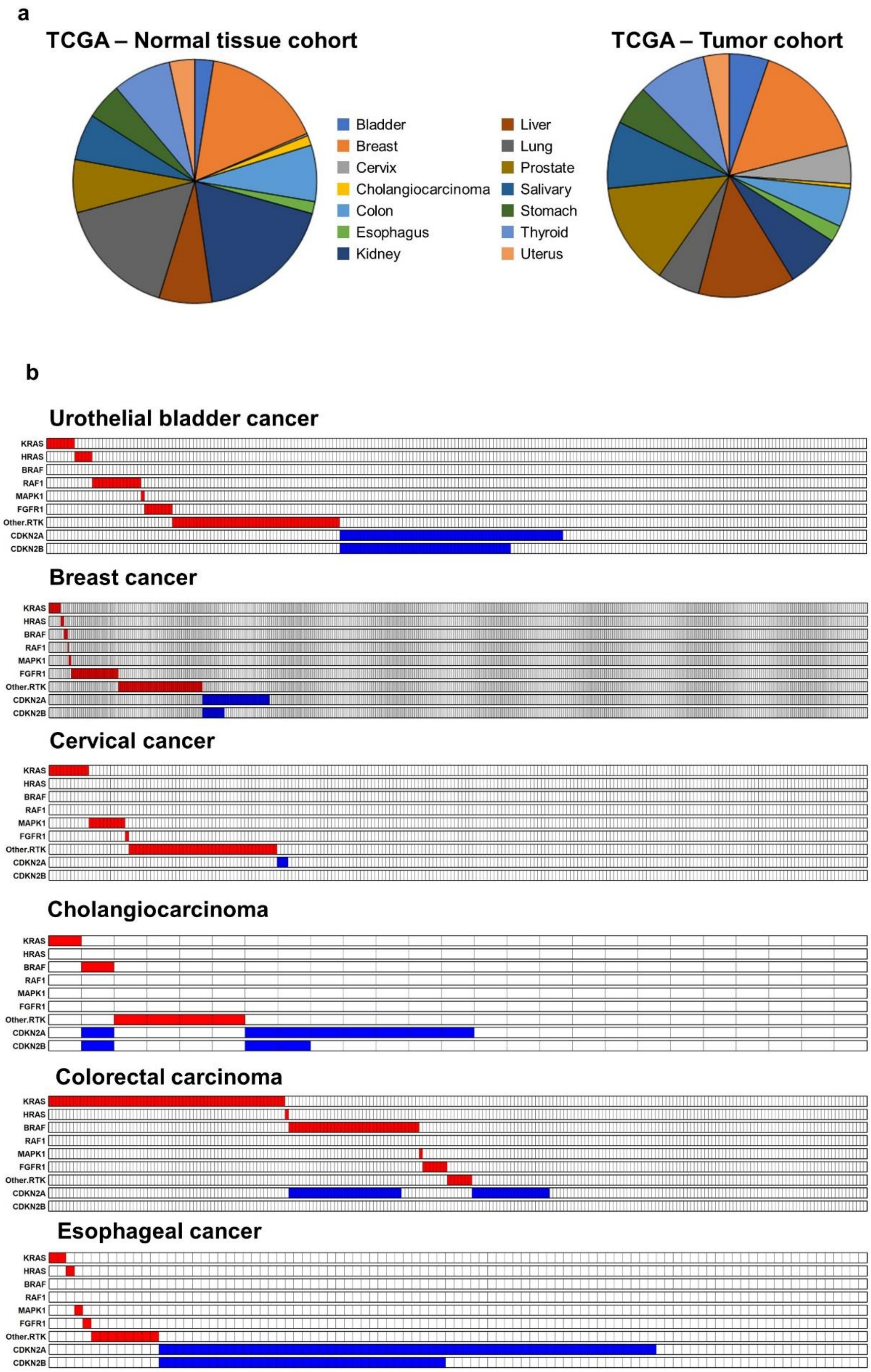
a
TCGA – Normal tissue cohort
TCGA – Tumor cohort
Bladder
Breast
Cervix
Cholangiocarcinoma
Colon
Esophagus
Kidney
Liver
Lung
Prostate
Salivary
Stomach
Thyroid
Uterus
b
Urothelial bladder cancer
Breast cancer
Cervical cancer
Cholangiocarcinoma
Colorectal carcinoma
Esophageal cancer
KRAS
HRAS
BRAF
RAF1
MAPK1
FGFR1
Other.RTK
CDKN2A
CDKN2B

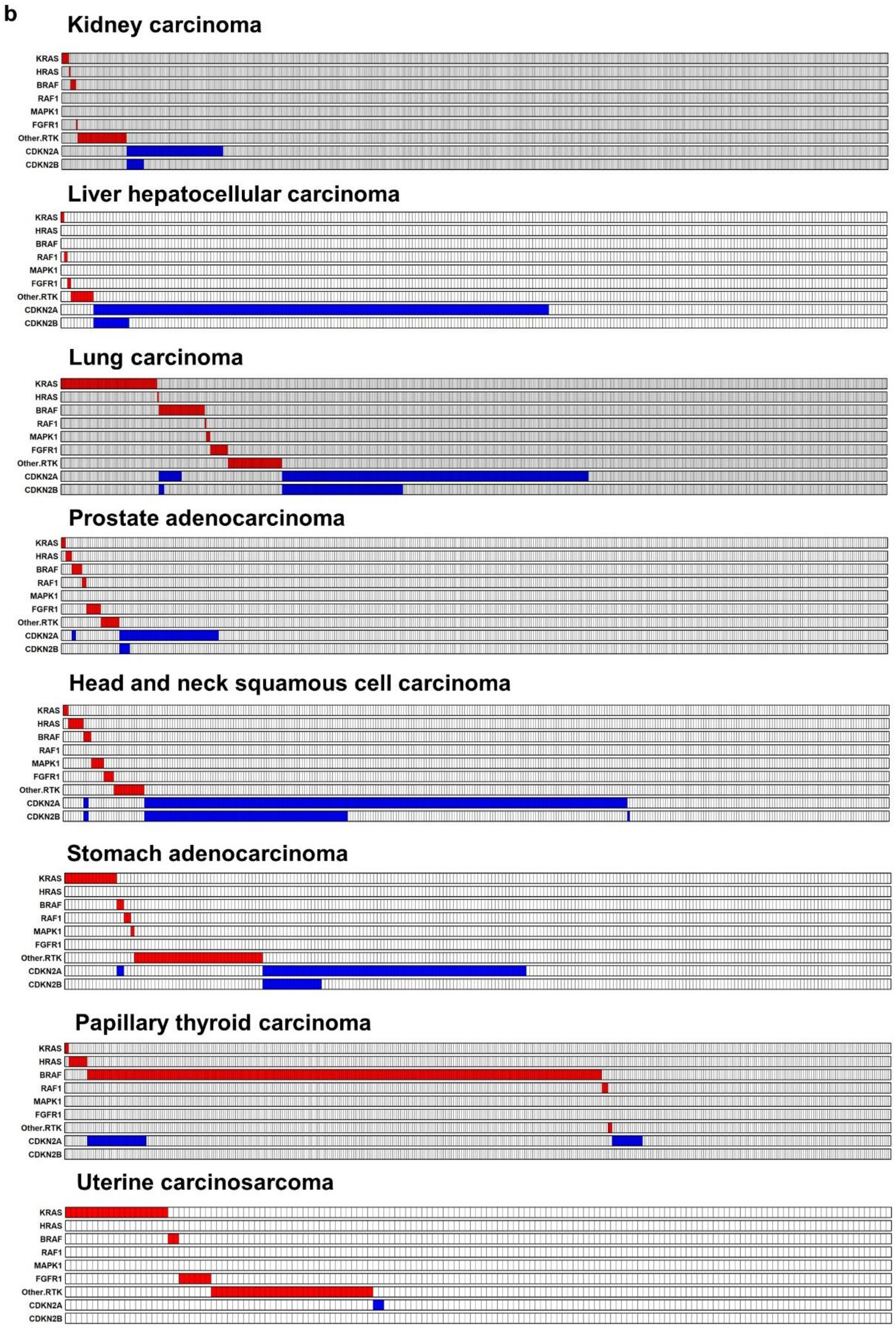
b
Kidney carcinoma
KRAS
HRAS
BRAF
RAF1
MAPK1
FGFR1
Other.RTK
CDKN2A
CDKN2B
Liver hepatocellular carcinoma
Lung carcinoma
Prostate adenocarcinoma
Head and neck squamous cell carcinoma
Stomach adenocarcinoma
Papillary thyroid carcinoma
Uterine carcinosarcoma

**Supplementary Fig. S12: Description of the TCGA dataset**

**a-b**, description of The Cancer Genomic Atlas (TCGA) cohort, regrouping several primary tumor and normal tissue samples from different entities and MAPK alteration backgrounds. The TCGA was pre-sorted to only keep samples with mutually exclusive MAPK alteration.

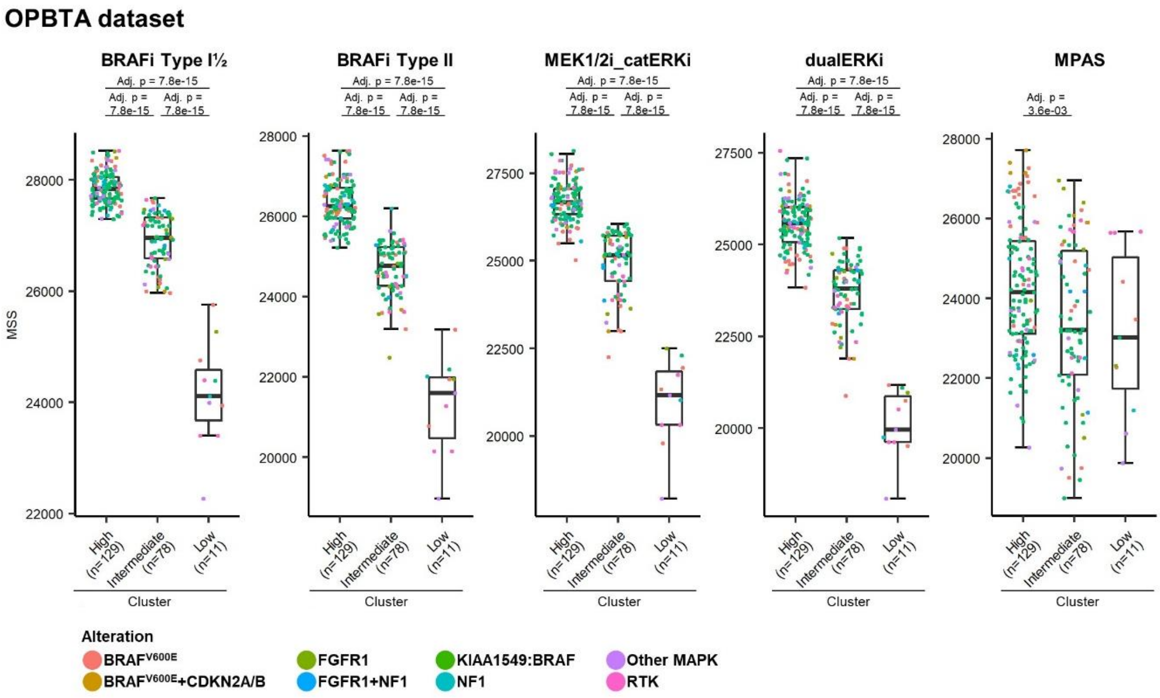


**Supplementary Fig. S13: Raw ssGSEA MAPKi sensitivity score in the identified sensitivity clusters in the pLGG samples from the OPBTA cohort**

Boxplots depicting raw ssGSEA MAPKi sensitivity scores and MPAS in the pLGG clusters from Fig. 4C from the OPBTA cohort. Dots are colored based on the detected driving MAPK alteration. Data from n = 218 biologically independent samples were used. Boxplots depict the median, first and third quartiles. Whiskers extend from the hinge to the largest/smallest value no further than 1.5 * IQR from the hinge (where IQR is the inter-quartile range). Significance was calculated using one-way ANOVA followed by the Tukey's 'Honest Significant Difference'. Asterisks depict significance as follows: * p-val < 0.05, ** p-val < 0.01, *** p-val < 0.001, not significant if not specified. Source data are provided as a Source Data file.

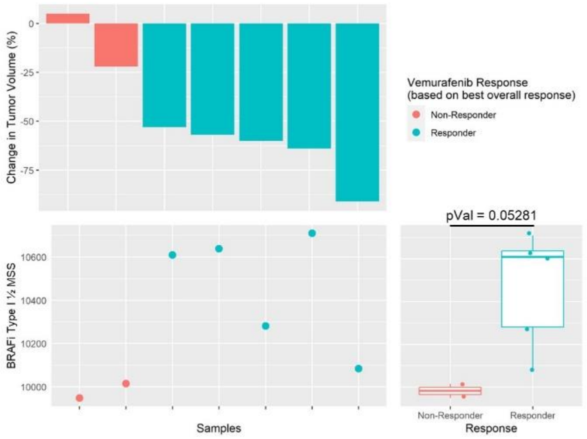


**Supplementary Fig. S14: Independent validation of MSSs in a melanoma cohort with RNAseq data at baseline coupled with vemurafenib response**

Waterfall plots depicting vemurafenib treatment response for each samples, and dotplots were used to depict the BRAFi Type I ½. Samples were grouped based on treatment response in boxplots (tumor volume reduction > 30% = responders; tumor volume reduction < 30% or progression = non-responders, as defined in the original article). Boxplots depict the median, first and third quartiles. Whiskers extend from the hinge to the largest/smallest value no further than 1.5 * IQR from the hinge (where IQR is the inter-quartile range). P-value was calculated using a Kruskal-Wallis rank sum test. Data from n = 7 biologically independent animals were used. Source data are provided as a Source Data file.

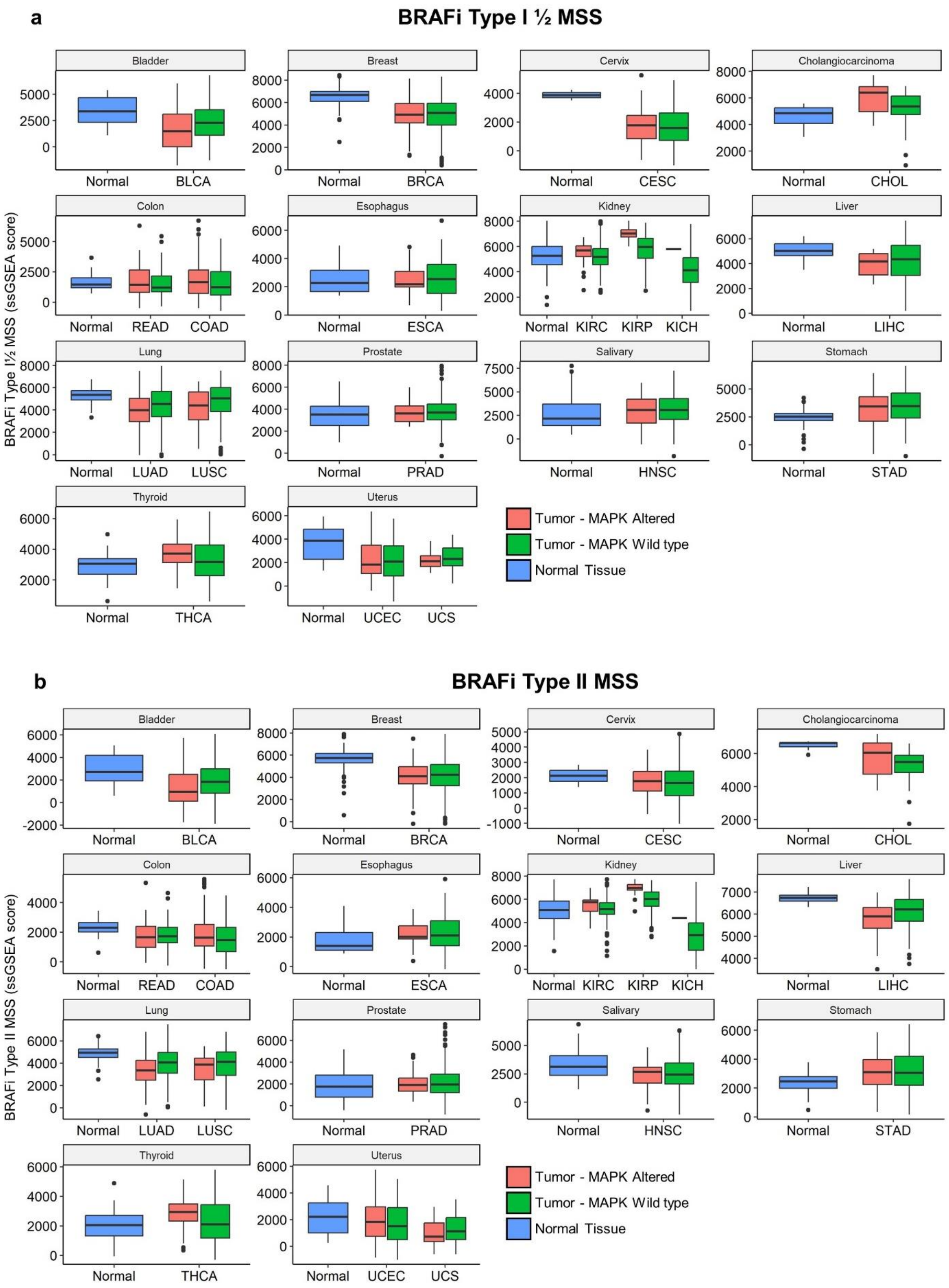

a
BRAFi Type I ½ MSS
BRAFi Type I½ MSS (ssGSEA score)
Bladder
Breast
Cervix
Cholangiocarcinoma
Colon
Esophagus
Kidney
Liver
Lung
Prostate
Salivary
Stomach
Thyroid
Uterus
Normal
BLCA
BRCA
CESC
CHOL
READ
COAD
ESCA
KIRC
KIRP
KICH
LIHC
LUAD
LUSC
PRAD
HNSC
STAD
THCA
UCEC
UCS
Tumor - MAPK Altered
Tumor - MAPK Wild type
Normal Tissue
b
BRAFi Type II MSS
BRAFi Type II MSS (ssGSEA score)
Bladder
Breast
Cervix
Cholangiocarcinoma
Colon
Esophagus
Kidney
Liver
Lung
Prostate
Salivary
Stomach
Thyroid
Uterus
Normal
BLCA
BRCA
CESC
CHOL
READ
COAD
ESCA
KIRC
KIRP
KICH
LIHC
LUAD
LUSC
PRAD
HNSC
STAD
THCA
UCEC
UCS
Tumor - MAPK Altered
Tumor - MAPK Wild type
Normal Tissue

**c** **MEK1/2i_dualERKi MSS**

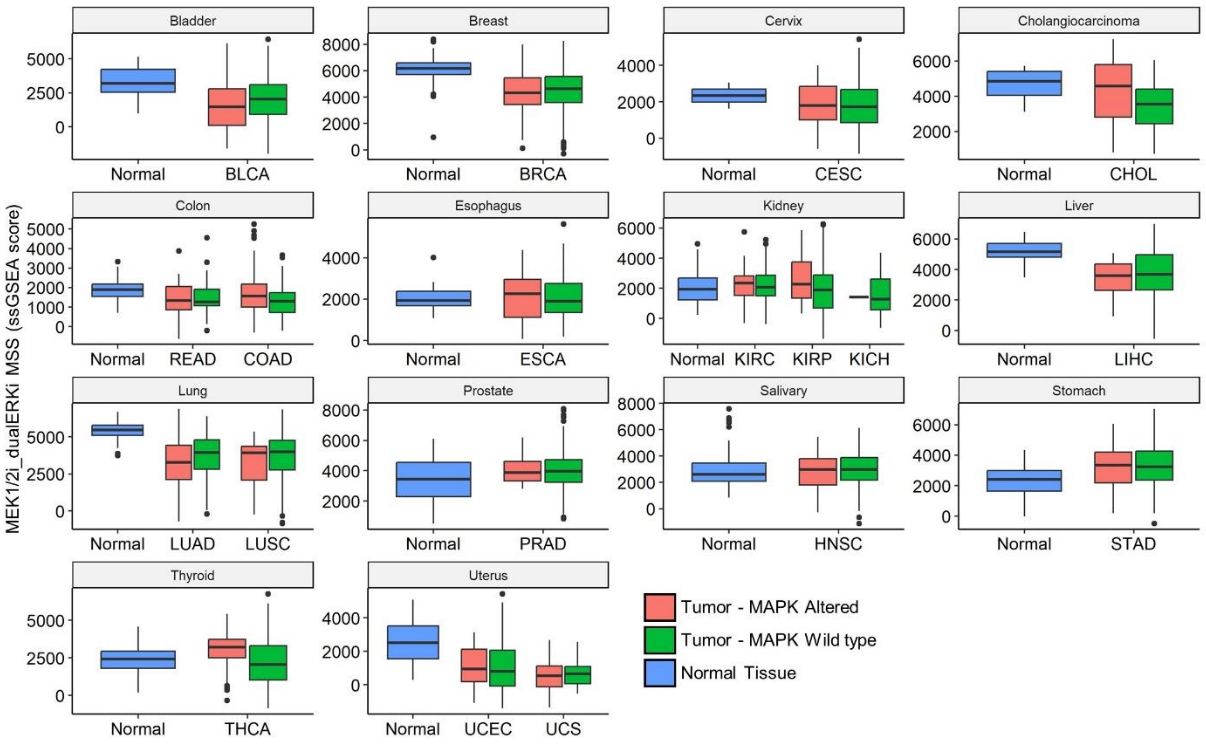


**d** **catERKi MSS**

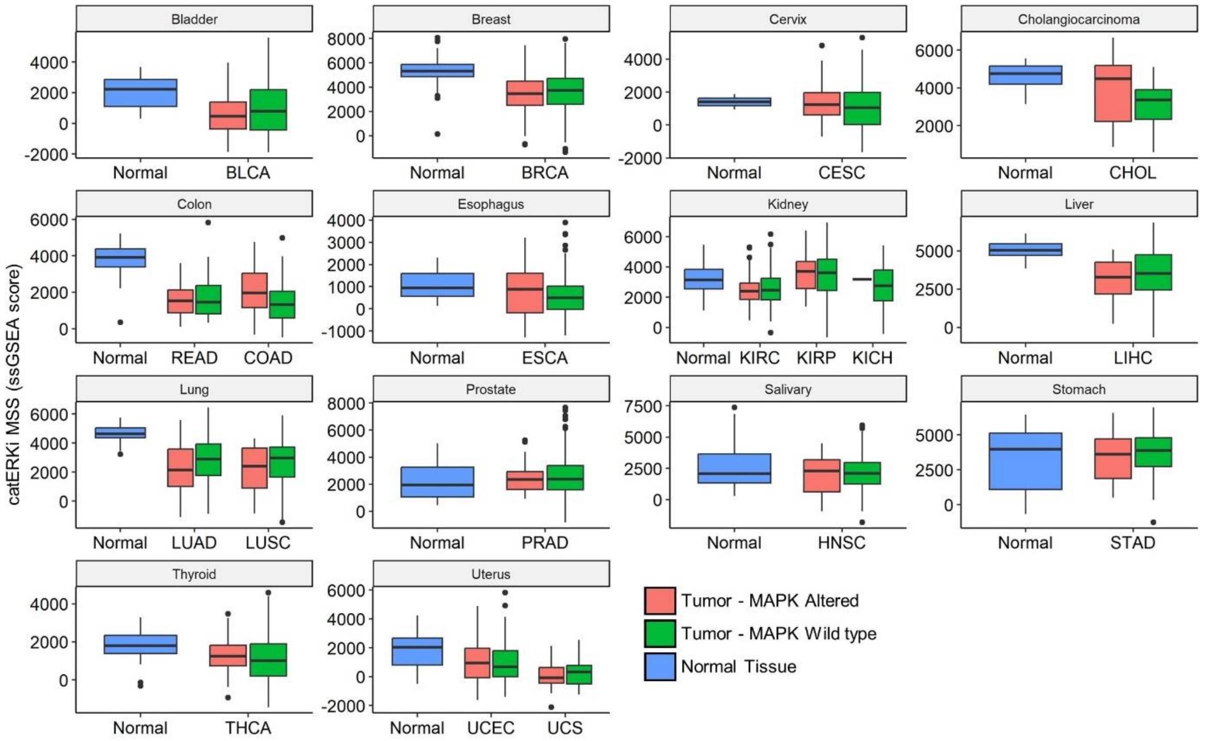

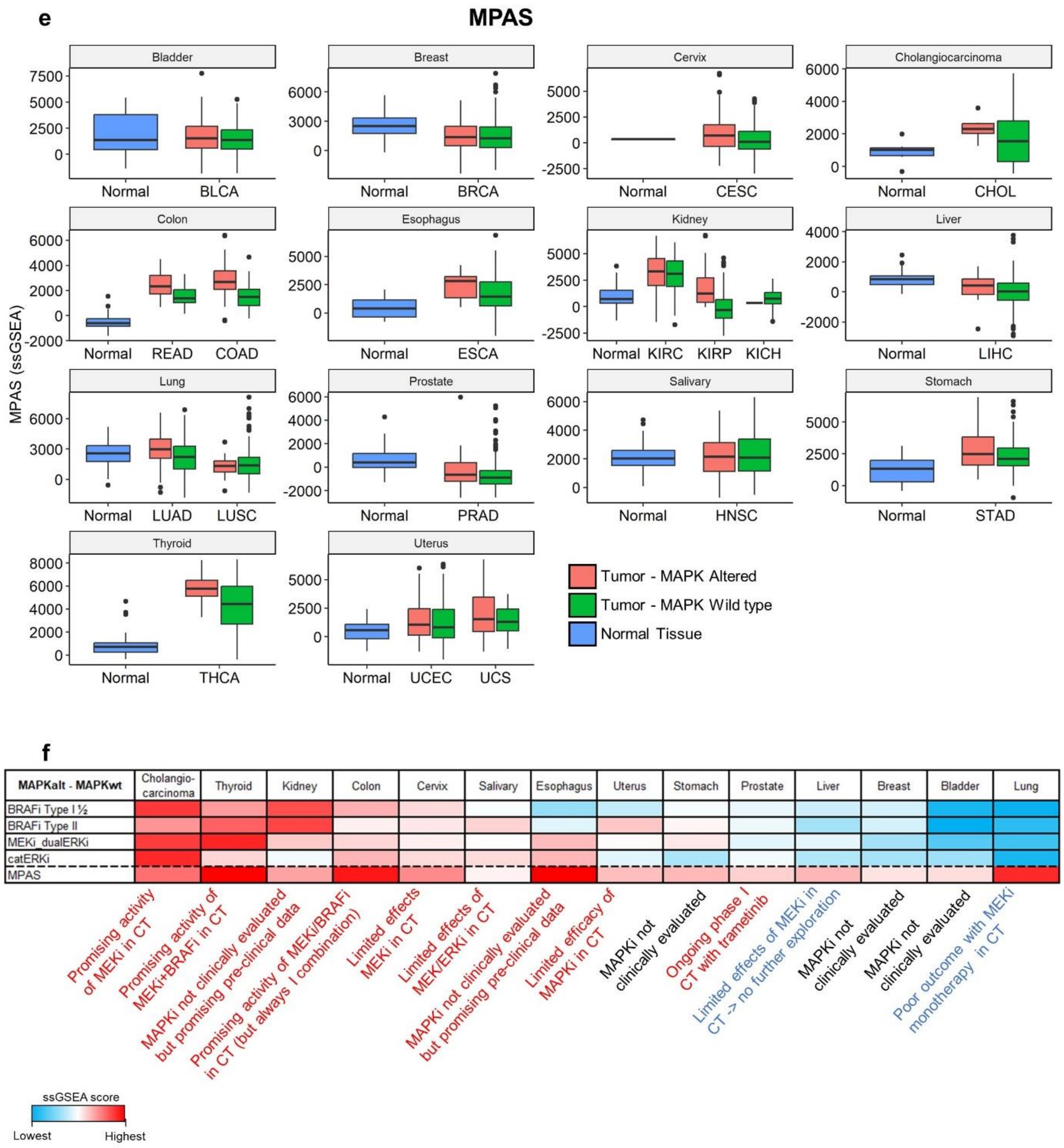


**Supplementary Fig. S15: Predicted MAPKi sensitivity scores in TCGA (normal and tumor) dataset**

**a-e**, boxplots showing raw ssGSEA score for the BRAFi Type I ½ (**a**), type II (**b**), MEK1/2i_catERKi (**c**), dual_ERKi (**d**) and MPAS (**e**) signatures in several non-CNS tumor entities and their corresponding normal tissue from the TCGA dataset. Boxplots depict the median, first and third quartiles. Whiskers extend from the hinge to the largest/smallest value no further than 1.5 * IQR from the hinge (where IQR is the inter-quartile range). Data from n = 5165 independent biological samples were used (see Source Data for details per tumor/tissue). **f**, summary heatmap summarizing the

signature score difference between MAPK altered vs MAPK wild type, and between tumor vs normal tissue. Blue colors indicate scores higher in MAPK wild type/normal tissue, while red colors indicate scores higher in MAPK altered/tumor samples. Bellow the heatmap is the status of MAPKi testing in the given entities, based a non-exhaustive literature review. Red writing indicates positive outcome in clinical trial, while blue indicates negative outcome in clinical trial. Black indicates no testing in clinical trial. Source data are provided as a Source Data file.

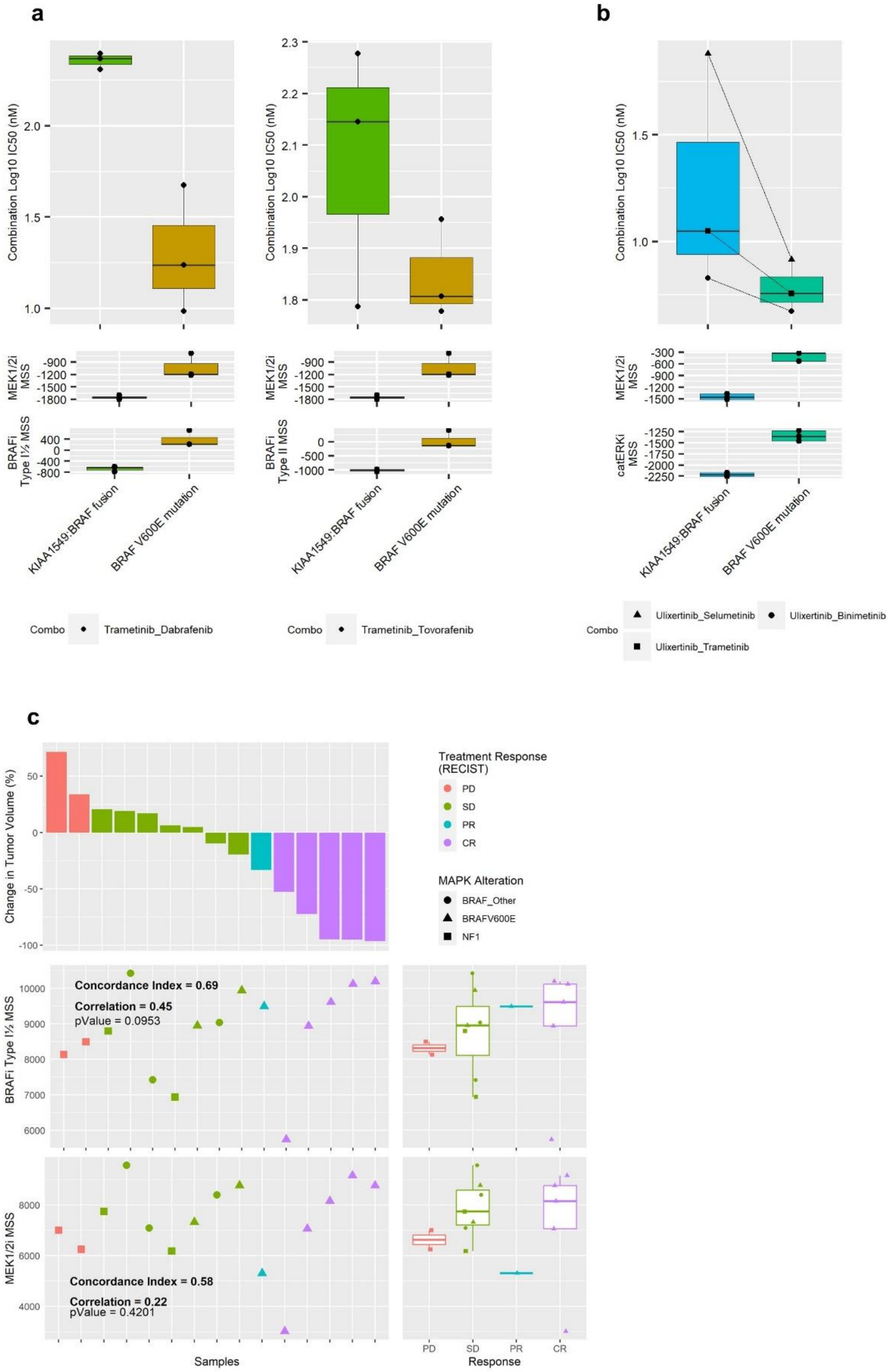

a
b
c
Combination Log10 IC50 (nM)
MEK1/2i MSS
BRAFi Type I½ MSS
BRAFi Type II MSS
catERKi MSS
KIAA1549:BRAF fusion
BRAF V600E mutation
Combo
Trametinib_Dabrafenib
Trametinib_Tovorafenib
Ulixertinib_Selumetinib
Ulixertinib_Binimetinib
Ulixertinib_Trametinib
Change in Tumor Volume (%)
Treatment Response (RECIST)
PD
SD
PR
CR
MAPK Alteration
BRAF_Other
BRAFV600E
NF1
Concordance Index = 0.69
Correlation = 0.45
pValue = 0.0953
Concordance Index = 0.58
Correlation = 0.22
pValue = 0.4201
Samples
Response

**Supplementary Fig. S16: Correlation between MAPKi sensitivity scores and MAPKi combination response *in vitro* and *in vivo***

**a, b**, matching analysis was then done between MAPKi sensitivity data has measured from a MAPK-reporter assay and the corresponding MSS for the BT40, DKFZ-BT66, DKFZ-BT314 and DKFZ-BT317 cell lines to explore each class-based in the context of a MAPKi therapy. Cells with a KIAA1549:BRAFfusion (DKFZ-BT317) and a $BRAF^{V600E}$ mutation (DKFZ-BT314) were treated with a combination of MEKi (trametinib) + BRAFi Type I½ (dabrafenib), and a combination of MEKi (trametinib) + BRAFi Type II (tovorafenib). Each dot represents a biological replicate (**a**). Data from a previously published dataset where cells with a KIAA1549:BRAFfusion (DKFZ-BT66) and a $BRAF^{V600E}$ mutation (BT40) were treated with a combination of a catERKi (ulixertinib) + MEKi (trametinib, binimetinib, or selumetinib). Each dot represents the average log10($IC_{50}$) from three biological replicates for each combinations (**b**). The combination log10 $IC_{50}$ was estimated by the $IC_{50}$ of the dose-response curve obtained when combining both drugs at their respective $IC_{50}$. **c**, waterfall plots depicting combination treatment response for each samples, and dotplots were used to depict corresponding MAPKi sensitivity scores. Samples were grouped based on treatment response in boxplots (i.e. primary response as described in the original publication; mRECIST criteria). Boxplots depict the median, first and third quartiles. Whiskers extend from the hinge to the largest/smallest value no further than 1.5 * IQR from the hinge (where IQR is the inter-quartile range). P-value was calculated using a Kruskal-Wallis rank sum test. Data from n = 15 independent animals were used. Source data are provided as a Source Data file.

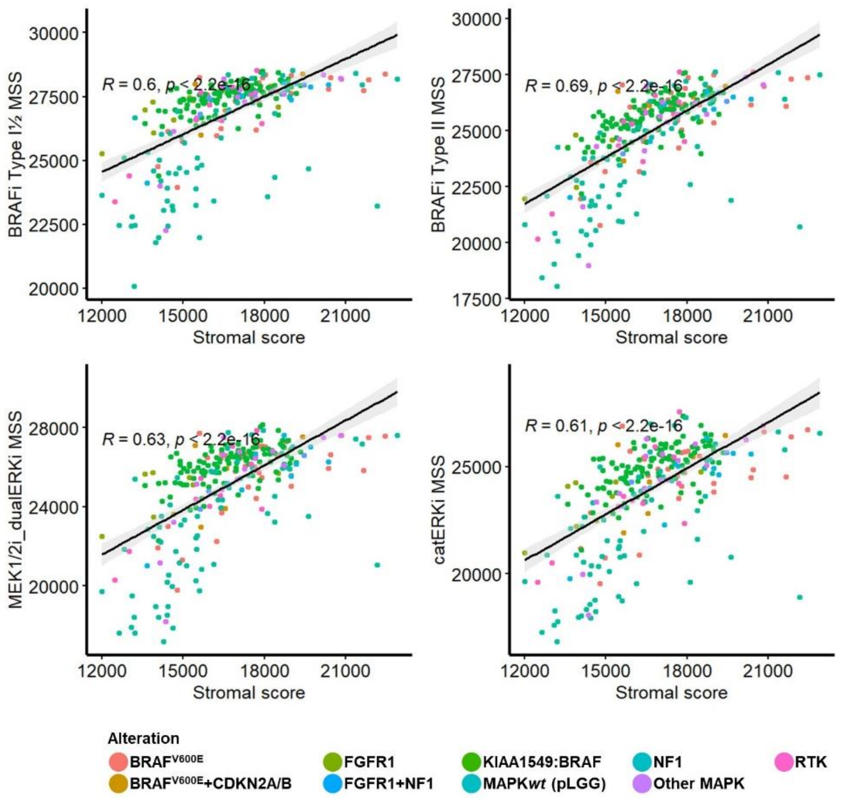


**Supplementary Fig. S17: Correlation between MAPKi sensitivity scores and stromal score in the pLGG samples from the OPBTA dataset**

Dotplots showing the correlation between the MAPKi sensitivity scores and the predicted stromal infiltration from the pLGG samples from the OPBTA dataset. Pearson's coefficient of correlation, corresponding p-value (two-tailed t-test), and the 95% confidence interval (error band) are depicted. Dots are colored based on the detected MAPK alteration. Source data are provided as a Source Data file.

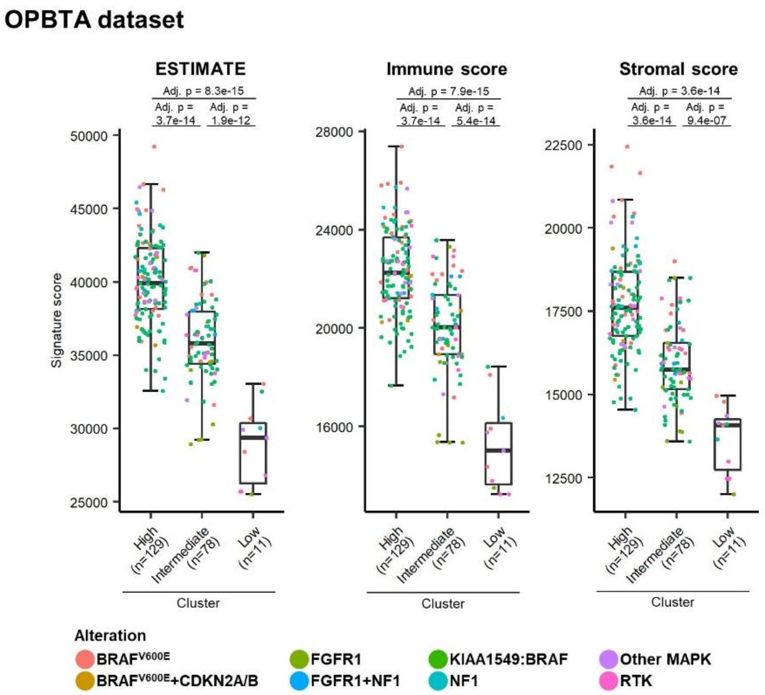


**Supplementary Fig. S18: Raw ssGSEA ESTIMATE score in the identified sensitivity clusters in the pLGG samples from the OPBTA cohort**

Boxplots depicting raw ssGSEA scores for the ESTIMATE and its signatures (immune and stromal) in the pLGG clusters from Fig. 4C from the OPBTA cohort. Dots are colored based on the detected driving MAPK alteration. Data from n = 218 biologically independent samples were used. Boxplots depict the median, first and third quartiles. Whiskers extend from the hinge to the largest/smallest value no further than 1.5 * IQR from the hinge (where IQR is the inter-quartile range). Significance was calculated using one-way ANOVA followed by the Tukey's ‘Honest Significant Difference’. Asterisks depict significance as follows: * p-val < 0.05, ** p-val < 0.01, *** p-val < 0.001, not significant if not specified. Source data are provided as a Source Data file.

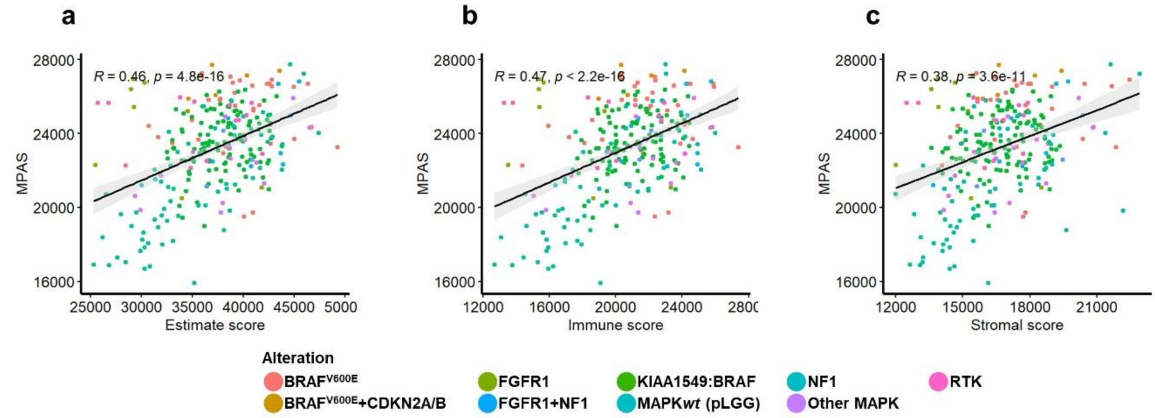


**Supplementary Fig. S19: Correlation between the MPAS and the ESTIMATE signature scores in the pLGG samples from the OPBTA dataset**

**a-c**, dotplots depict the correlation between the MPAS and the ESTIMATE (**a**), predicted immune infiltration (**b**) and predicted stromal infiltration (**c**) in the pLGG samples from the OPBTA. Pearson's coefficient of correlation, corresponding p-value (two-tailed t-test), and the 95% confidence interval (error band) are depicted. Dots are colored based on the detected MAPK alteration. Source data are provided as a Source Data file.

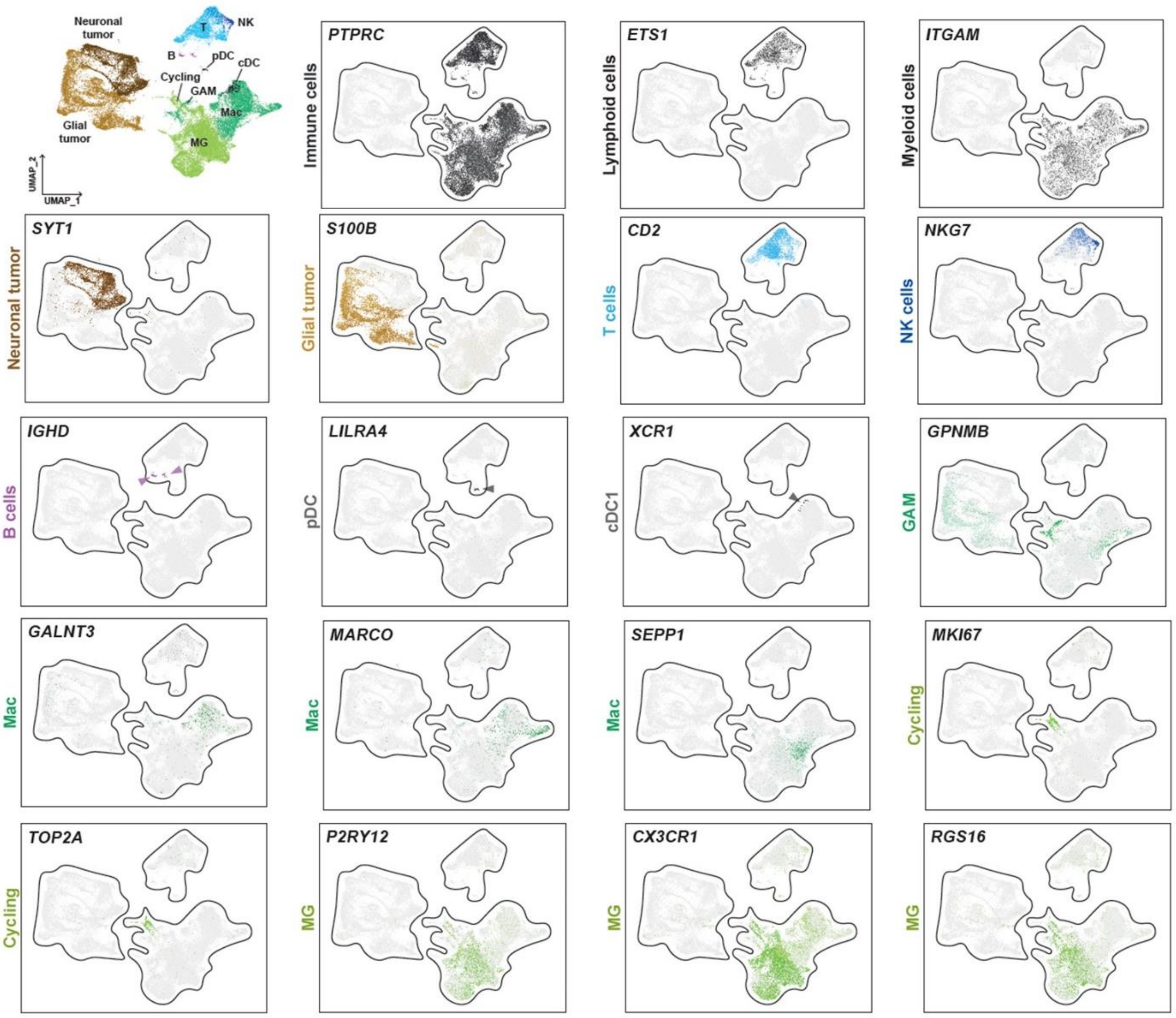


283

284 **Supplementary Fig. S20: Characterization of cell population from pLGG samples using scRNA**
285 **sequencing**
286 Supplementary UMAPs depicting key markers for tumor, lymphoid and myeloid cell populations and
287 subpopulations.

288

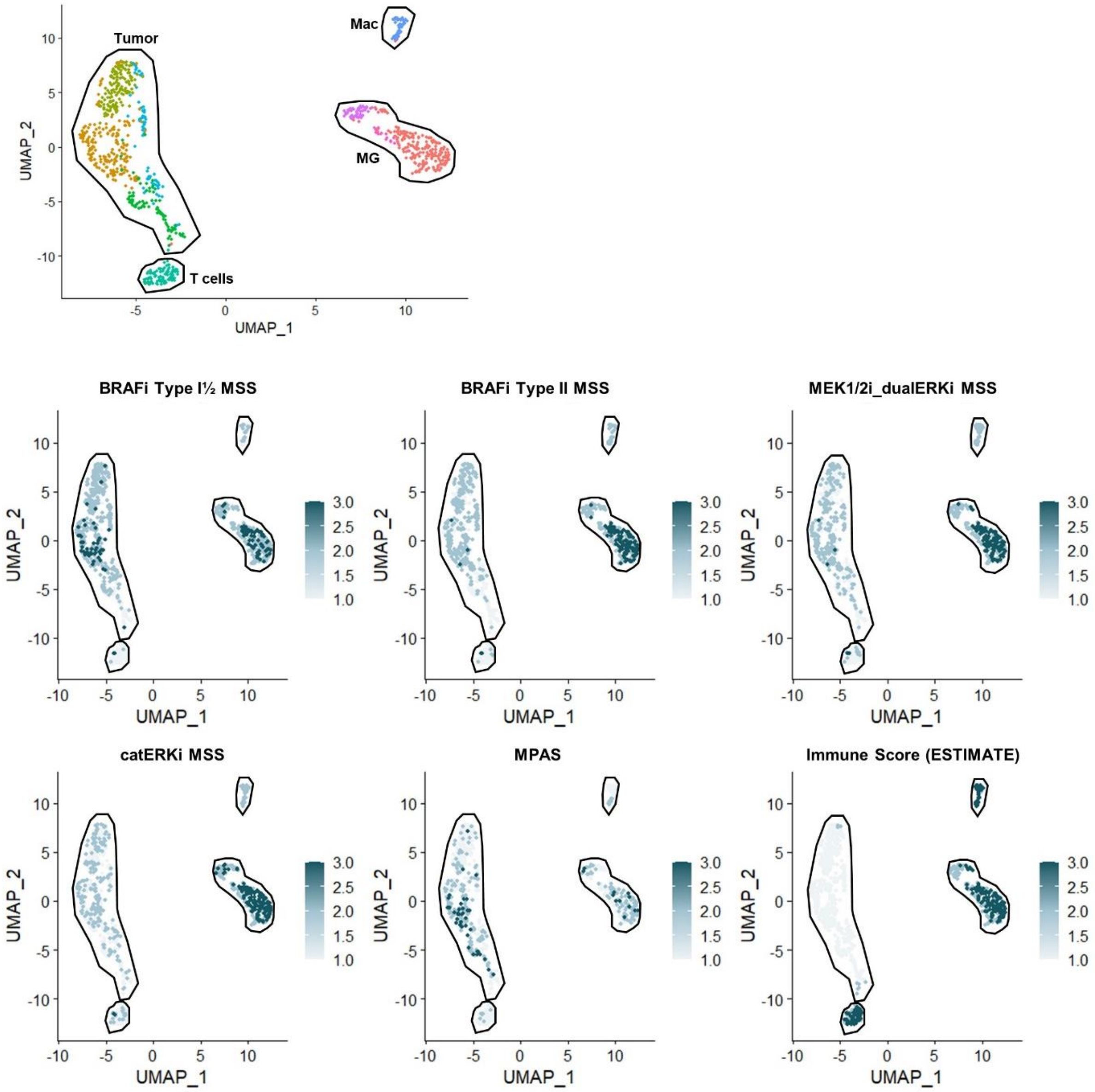


**Supplementary Fig. S21: MSS in microglia from scRNAseq dataset from Reitman et al.**
UMAPs depicting the key cell populations identified in the Reitman's dataset (as annotated in the original publication), and the respective MSS, MPAS and immune score.

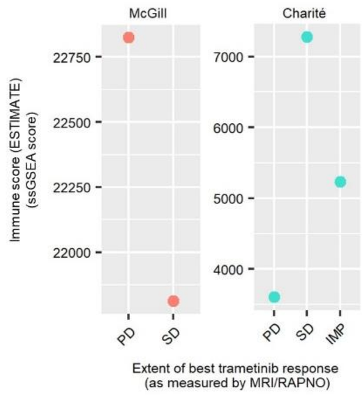


**Supplementary Fig. S22: Immune score (ESTIMATE) in pLGG samples from baseline with response to MEKi treatment**

Dotplot depicting the predicted immune infiltration (ESTIMATE) in pLGG derived primary samples from patients who received a trametinib treatment. Gene expression was measured on samples acquired prior treatment initiation. Since the MSS are not comparable across datasets, the samples were split based on institute of origin. Source data are provided as a Source Data file.

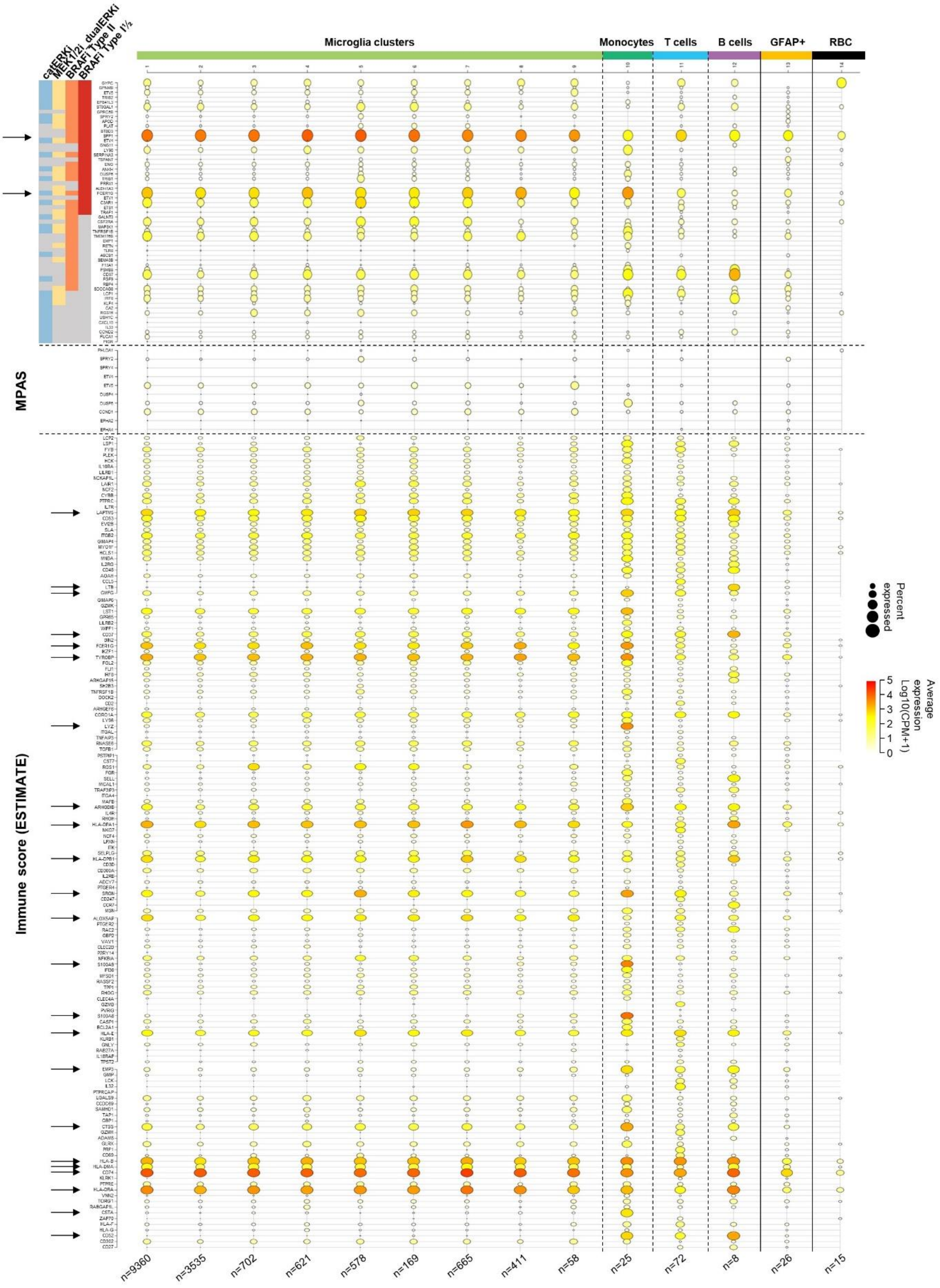

calERKi
MEK1/2i, dualERKi
BRAFi Type II
BRAFi Type I½
Microglia clusters
Monocytes
T cells
B cells
GFAP+
RBC
MPAS
Immune score (ESTIMATE)
Percent expressed
Average expression Log10(CPM+1)
n=9360
n=3535
n=702
n=621
n=578
n=169
n=665
n=411
n=58
n=25
n=72
n=8
n=26
n=15

**Supplementary Fig. S23: MSS, MPAS and immune score (ESTIMATE) gene expression in non-tumor associated microglia**

Dotplot depicting average percentage expression across all cells with a cluster (circle size) and average expression level (color code) in each cellular clusters identified in scRNAseq analysis of samples from Alzheimer and epileptic patients. Arrows indicate highly expressed genes (log(CPM+1) > 2).

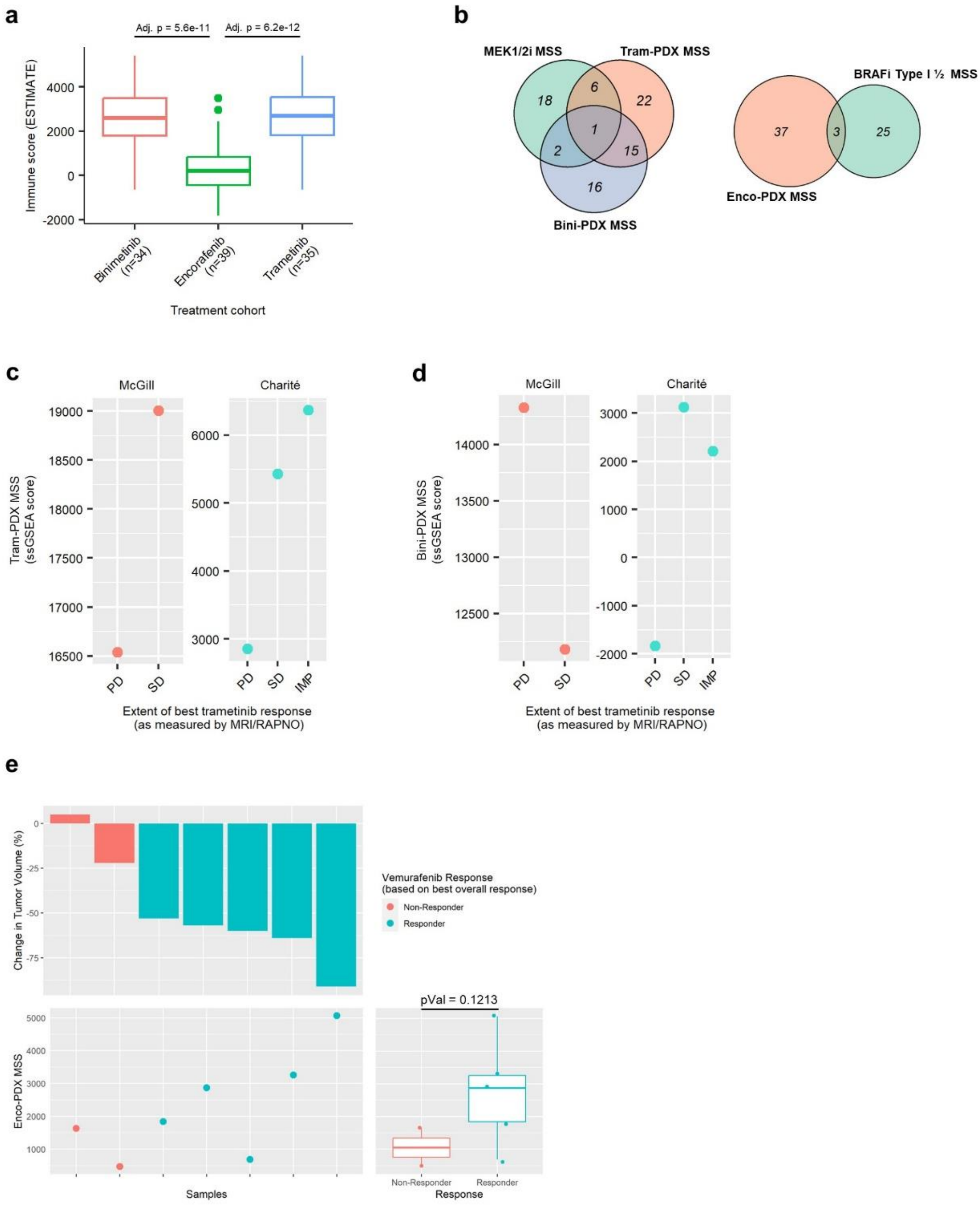


**Supplementary Fig. S24: Analysis of PDX-derived MSSs for the MEKi trametinib and binimetinib, and the BRAFi Type I ½ encorafenib**

**a**, bloxplot depicting the proportion of predicted immune infiltration (as measured with the ESTIMATE signature) in the PDX cohort treated with trametinib, binimetinib and encorafenib. Data from n = 108 biologically independent animals were used. Boxplots depict the median, first and third quartiles. Whiskers extend from the hinge to the largest/smallest value no further than 1.5 * IQR from the hinge (where IQR is the inter-quartile range). Significance was calculated using one-way ANOVA followed by the Tukey's ‘Honest Significant Difference’. **b**, Venn diagram depicting genes overlap between MSS

signatures derived from *in vitro* and *in vivo* (PDX from XevaDB) samples. **c-d**, Dotplot depicting the Tram-PDX MSS (**c**) and Bini-PDX MSS (**d**) in pLGG derived primary samples from patients who received a trametinib treatment. Gene expression was measured on samples acquired prior treatment initiation. Since the MSS are not comparable across datasets, the samples were split based on institute of origin. **e**, Waterfall plots depicting vemurafenib treatment response for each samples, and dotplots were used to depict the Enco-PDX MSS. Samples were grouped based on treatment response in boxplots (tumor volume reduction > 30% = responders; tumor volume reduction < 30% or progression = non-responders, as defined in the original article). Boxplots depict the median, first and third quartiles. Whiskers extend from the hinge to the largest/smallest value no further than 1.5 * IQR from the hinge (where IQR is the inter-quartile range). P-value was calculated using a Kruskal-Wallis rank sum test. Data from n = 7 biologically independent animals were used. Source data are provided as a Source Data file.

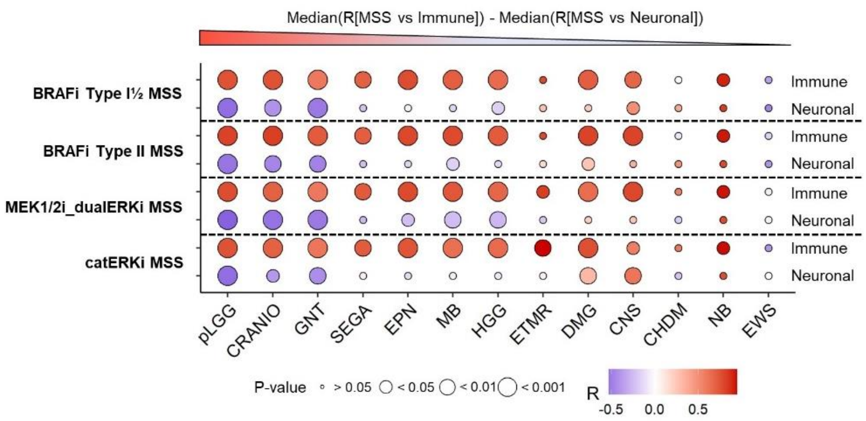


**Supplementary Fig. S25: Correlation analysis between the MSSs and the immune/neuronal scores in the OPBTA cohort**

Dotplot summarizing the correlation coefficient (R) when comparing the indicated MSS to the immune or neuronal score in the OPBTA dataset. The entities were split based on the difference between the median R from the correlation between MSS vs immune score, and median R from the correlation between MSS vs neuronal score. The medians were measure across the four MSSs. Dot size depicts the respective p-value (two-tailed t-test). pLGG: low-grade glioma; CRANIO: craniopharyngioma; GNT: glial neuronal tumor; SEGA: Subependymal Giant Cell Astrocytoma ; EPN: ependymoma; MB: medulloblastoma; HGG: high-grade glioma; ETMR: embryonal tumor with multilayer rosettes; DMG: diffuse midline glioma; CNS: other CNS embryonal tumor; CHDM: chordoma; NB: neuroblastoma; EWS: Ewin Sarcoma. Source data are provided as a Source Data file.
