# Supplementary material for: MAPK inhibitor sensitivity scores predict sensitivity driven by the immune infiltration in pediatric low-grade gliomas
Source: Nat Commun. 2023 Jul 27;14:4533. doi: 10.1038/s41467-023-40235-8 (PMC10374577; doi:10.1038/s41467-023-40235-8)
Supplement: Supplementary file 3 — Description of Additional Supplementary Files [file 41467_2023_40235_MOESM3_ESM.pdf]

Description of Additional Supplementary Files

Supplementary Data Legends

Supplementary Data 1: Sorted list of MAPKi used in the GDSC dataset

Supplementary Data 2: List of 9 signatures derived from Discovery set

Supplementary Data 3: List and values of metrics calculated for each MAPKi in both Discovery and Training sets

Supplementary Data 4: List of validated MSS signatures (n = 4), Overlap MSS signature, MPAS signature and PDX-derived MSS

Supplementary Data 5: MSS in pLGG - Melanoma - Multiple myeloma cell lines

Supplementary Data 6: pLGG patient cohorts' details

Supplementary Data 7: Multiple linear regression analysis of predictors associated with predicted MAPKi sensitivity

Supplementary Data 8: Drugs used in the MAPKi mini-screen in DKFZ-BT317_pDIPZ

Supplementary Data 9: Gene expression data, IC50 z-scores and signature scores for cell lines from Discovery and Testing sets

Supplementary Data 10: MAS5.0 normalized gene expression profiles from pLGG cell lines, multiple myeloma samples and melanoma samples from the R2 platform.

Supplementary Software legend

Supplementary Software: Supplementary R script
