# Supplementary material for: MAPK inhibitor sensitivity scores predict sensitivity driven by the immune infiltration in pediatric low-grade gliomas
Source: Nat Commun. 2023 Jul 27;14:4533. doi: 10.1038/s41467-023-40235-8 (PMC10374577; doi:10.1038/s41467-023-40235-8)
Supplement: Supplementary file 15 — Reporting Summary [file 41467_2023_40235_MOESM15_ESM.pdf]

nature portfolio

Corresponding author(s): Till Milde, Romain Sigaud

Last updated by author(s): Jul 13, 2023

Reporting Summary

Nature Portfolio wishes to improve the reproducibility of the work that we publish. This form provides structure for consistency and transparency in reporting. For further information on Nature Portfolio policies, see our Editorial Policies and the Editorial Policy Checklist.

Statistics

For all statistical analyses, confirm that the following items are present in the figure legend, table legend, main text, or Methods section.

n/a	Confirmed	
☐	☒	The exact sample size (n) for each experimental group/condition, given as a discrete number and unit of measurement
☐	☒	A statement on whether measurements were taken from distinct samples or whether the same sample was measured repeatedly
☐	☒	The statistical test(s) used AND whether they are one- or two-sided<br>*Only common tests should be described solely by name; describe more complex techniques in the Methods section.*
☐	☒	A description of all covariates tested
☐	☒	A description of any assumptions or corrections, such as tests of normality and adjustment for multiple comparisons
☐	☒	A full description of the statistical parameters including central tendency (e.g. means) or other basic estimates (e.g. regression coefficient) AND variation (e.g. standard deviation) or associated estimates of uncertainty (e.g. confidence intervals)
☐	☒	For null hypothesis testing, the test statistic (e.g. F, t, r) with confidence intervals, effect sizes, degrees of freedom and P value noted<br>*Give P values as exact values whenever suitable.*
☒	☐	For Bayesian analysis, information on the choice of priors and Markov chain Monte Carlo settings
☒	☐	For hierarchical and complex designs, identification of the appropriate level for tests and full reporting of outcomes
☐	☒	Estimates of effect sizes (e.g. Cohen's d, Pearson's r), indicating how they were calculated

Our web collection on statistics for biologists contains articles on many of the points above.

Software and code

Policy information about availability of computer code

Data collection	OPTIMA BMG-Labtech software (v2.20R2), GraphPad Prism 5 software (Version 5.01)

Data analysis

All statistical analysis were performed in R Studio (R Version 1.4.1103), using the following packages:
- Correlation analysis and Pearson coefficient were carried out using the "stats" package (v4.2.1).
- Concordance indices were calculated using the "survival" package (v3.4-0).
- Consensus ranking and related figures were done using the "challengeR" package (v1.0.2).103
- Signatures overlap and Venn diagrams were generated using the "VennDiagram" package (v1.7.3).
- Significance between groups was calculated using ANOVA followed by the Tukey's 'Honest Significant Difference' test in the "stats" package.
- Multiple linear regression analysis was performed with the lm function, after ensuring that the data followed a normal distribution (Shapiro-Wilk normality test).
- Gene Set Enrichment Analysis (GSEA) was performed using the Broad Institute software (GSEA_4.0.3).
- The ssGSEA module (v10.1.0) from Gene Pattern was used to measure ssGSEA scores, using the parameters recommended in the documentation. Of note, ssGSEA scores were not normalized, and are therefore considered as measured in arbitrary units.
- The testing of the extent to which our signatures are confounded due to the microenvironmental transcriptome was done using ConfoundR (https://confoundr.qub.ac.uk/).
- scRNAseq data was analyzed using the "Seurat" package in R (v4.3.0). scRNAseq data from Reitman et al. was re-analyzed with the "Seurat" package using the parameters described in the original publication, and signature scores were measured using the "UCell" package (v2.0.1).
Graphical representations were done in R Studio using the "ggplot2" package (v3.4.1) for the correlation plots, waterfall plots and boxplots.
The "ComplexHeatmap" package (v2.12.1) was used to generate the heatmaps.

The custom R script used to select the best predicting signature can be found in the Supplementary Information file.

For manuscripts utilizing custom algorithms or software that are central to the research but not yet described in published literature, software must be made available to editors and reviewers. We strongly encourage code deposition in a community repository (e.g. GitHub). See the Nature Portfolio guidelines for submitting code & software for further information.

Data

Policy information about availability of data

All manuscripts must include a data availability statement. This statement should provide the following information, where applicable:

- Accession codes, unique identifiers, or web links for publicly available datasets
- A description of any restrictions on data availability
- For clinical datasets or third party data, please ensure that the statement adheres to our policy

Gene expression data (RMA normalized expression data) can be accessed from the GDSC website (https://www.cancerrxgene.org/gdsc1000/GDSC1000_WebResources/Home.html). Cell lines' drug response and genetic features can be download at the following link (https://www.cancerrxgene.org/downloads/anova).
Gene expression data and drug response in from the Novartis PDX cohort can be accessed at the following address: https://www.xevadb.ca/ and in the Supplementary information from the original publication (PMID: 26479923).
The already published gene expression profiles from pLGG cell lines, melanoma (GSE7127, [https://www.ncbi.nlm.nih.gov/geo/query/acc.cgi?acc=GSE7127]) and multiple myeloma (GSE6205, [https://www.ncbi.nlm.nih.gov/geo/query/acc.cgi?acc=GSE6205]) can be accessed on the R2 platform [https://hgserver1.amc.nl/cgi-bin/r2/main.cgi] (see also Suppl. Dataset 1 for MAS5.0 normalized data).
Processed genomic data from then Open Pediatric Brain Tumor Atlas dataset is available through the Open Pediatric Brain Tumor Atlas portal [https://github.com/AlexsLemonade/OpenPBTA-analysis]. Finally, unified TCGA sequencing data is accessible on figshare [https://figshare.com/articles/dataset/Data_record_3/5330593] and mutation status was retrieved from already published work.
Publicly available RNA sequencing datasets from melanoma primary samples with MAPKi response105 can be accessed from the GEO platform (GSE65185, [https://www.ncbi.nlm.nih.gov/geo/query/acc.cgi?acc=GSE65185]). Gene expression from pediatric SEGA samples with TSC1/2 mutation can be accessed from the publication and the European Genome-phenome Archive (EGAS00001003787, [https://ega-archive.org/studies/EGAS00001003787]).
The RNA sequencing data from our pLGG primary samples with trametinib response (GSE222406, [https://www.ncbi.nlm.nih.gov/geo/query/acc.cgi?acc=GSE222406]) and scRNA sequencing data from n = 6 pLGG samples (GSE222850, [https://www.ncbi.nlm.nih.gov/geo/query/acc.cgi?acc=GSE222850] generated in this study are both available from the GEO platform. All RAW data are available.
Source data are provided with this paper.

Human research participants

Policy information about studies involving human research participants and Sex and Gender in Research.

Reporting on sex and gender	Sex was not a criteria for study design or data interpretation. This information was not collected.
Population characteristics	The patient's clinicopathological characteristics are summarized in Supplementary Data S6.
Recruitment	Patient samples were colleted from archival storage. Patients were not actively recruited for this study.
Ethics oversight	Legal guardians provided written informed consent on behalf of all pediatric patients for the use of tissues for research without compensation. All samples from McGill collaborators (CHU Sainte-Justine biobank) were collected under protocol approved by the ethical commitee of CHU Sainte-Justine. All samples from Charité (archives from the SIOP-LGG 2004 interim protocol) were collected under protocol approved by the ethical commitee of Charité Universitätmedizin Berlin.

Note that full information on the approval of the study protocol must also be provided in the manuscript.

Field-specific reporting

Please select the one below that is the best fit for your research. If you are not sure, read the appropriate sections before making your selection.

☒ Life sciences ☐ Behavioural & social sciences ☐ Ecological, evolutionary & environmental sciences

For a reference copy of the document with all sections, see nature.com/documents/nr-reporting-summary-flat.pdf

Life sciences study design

All studies must disclose on these points even when the disclosure is negative.

Sample size	For all in vitro experiments carried out in this study, experiments were carried out in 3 independent biological replicates, as standard practise. Bulk RNA seq was performed on n = 5 samples, and scRNA seq was performed on n = 6 samples, as these were the only patient samples available at the time of the study.<br>The rest of the analyses were carried out on publicly available datasets.
Data exclusions	Data were excluded from the publicly available datasets used in the study based on criteria defined beforehand. These criteria were chosen to stay relevant to pediatric low-grade glioma biology (i.e. samples/cell lines with mutually exclusive genetic MAPK-alteration), and to stay clinically relevant (i.e. exclusion of data generated from drugs other than MAPK inhibitors, or MAPK inhibitors without clear mode of action/ already excluded from clinical trials).
Replication	All measures performed experimentally were always done in three independent biological replicates. All replicates are shown in the manuscript. All attempts at replication were successful.
Randomization	For in vitro experiments, cells were randomly allocated into control and experimental groups.
Blinding	Blinding was not applicable to the in vitro experiments because the same investigator was doing group allocation during data collection and/ or analysis.

Reporting for specific materials, systems and methods

We require information from authors about some types of materials, experimental systems and methods used in many studies. Here, indicate whether each material, system or method listed is relevant to your study. If you are not sure if a list item applies to your research, read the appropriate section before selecting a response.

Materials & experimental systems

n/a	Involved in the study	
☒	☐	Antibodies
☐	☒	Eukaryotic cell lines
☒	☐	Palaeontology and archaeology
☒	☐	Animals and other organisms
☒	☐	Clinical data
☒	☐	Dual use research of concern

Methods

n/a	Involved in the study	
☒	☐	ChIP-seq
☒	☐	Flow cytometry
☒	☐	MRI-based neuroimaging

Eukaryotic cell lines

Policy information about cell lines and Sex and Gender in Research

Cell line source(s)	The following cell lines were generated in our previous work:<br>DKFZ-BT66 were derived from a pilocytic astrocytoma from a 2-year old male patient (PMID: 28002790).<br>DKFZ-BT308 were derived from a pilocytic astrocytoma from a 5-year old male patient (PMID: 35977048).<br>DKFZ-BT314 were derived from a pilocytic astrocytoma from a 3-month old female patient (PMID: 35977048).<br>DKFZ-BT317 were derived from a pilocytic astrocytoma from a 2-year old male patient (PMID: 35977048).<br>BT40 were derived from a Juvenile Pilocytic Astrocytoma, and kindly provided by Prof. Peter Houghton.<br>Sex and gender were not considered in this study.
Authentication	Cell lines were authenticated using Multiplex Cell Authentication (Single Nucleotide Polymorphism profiling or STR profiling), and purity was validated using the Multiplex cell Contamination Test, both performed by Multiplexion (Heidelberg, Germany).
Mycoplasma contamination	Cell lines were tested negative for mycoplasma contamination.
Commonly misidentified lines<br>(See ICLAC register)	No commonly misidentified cell lines were used in this study.
